# Synthesis of Phthalides and α,β-butenolides by Transition Metal-Catalyzed Activation of C—H Bonds

**DOI:** 10.3390/molecules24040824

**Published:** 2019-02-25

**Authors:** Andrea Renzetti, Kozo Fukumoto

**Affiliations:** Faculty of Education, University of the Ryukyus, 1 Senbaru, Nishihara, Okinawa 903-0213, Japan; k-fuku@edu.u-ryukyu.ac.jp

**Keywords:** heterocycle, phthalide, butenolide, C—H activation, coupling, lactonization

## Abstract

Phthalides and α,β-butenolides are two related classes of oxygenated heterocycles with a wide range of biological activities. An innovative strategy to prepare these compounds is based on C—H bond functionalization reactions, in which two simple, unfunctionalized molecules are coupled together with cleavage of a C—H bond and formation of a C—X bond (X=C or heteroatom). This paper reviews the methods for the synthesis of phthalides and α,β-butenolides by C—H bond functionalization from non-halogenated starting materials. Over 30 methods are reported, mostly developed during the past ten years.

## 1. Introduction

**Figure 1:**
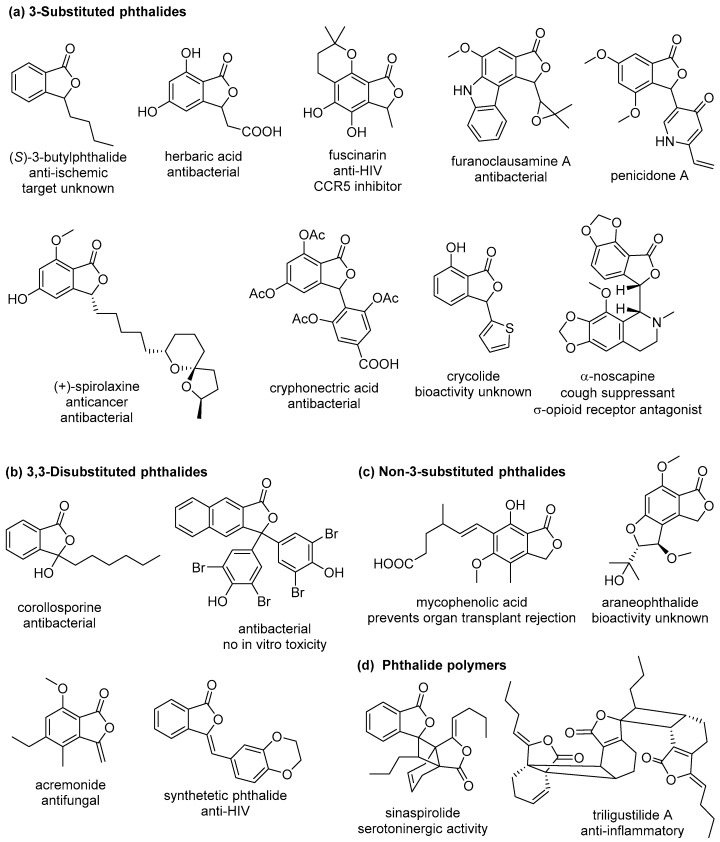
Representative examples of biologically active phthalides.

Phthalides (isobenzofuranones) are a family of heterocyclic compounds characterized by the presence of a benzene ring fused to a γ-lactone. They occur naturally in plants, fungi, and lichens, and display a wide range of biological activities such as antimicrobial, antioxidant, antiplatelet, and analgesic [1,2,3]. Herbal remedies containing phthalides are widely used in traditional medicine [4]; and some phthalides are marketed in the pure form as drugs. For instance, *n*-butylphthalide, which is responsible for the characteristic odor of celery, is on the market as an antiplatelet drug for the treatment of cerebral ischemia [5]; mycophenolic acid is a drug used for the prevention of organ transplant rejection [6]. Besides therapy, phthalides find application as synthetic intermediates [6], dyes (such as phenolphthalein), and flavoring agents. Phthalides can be classified into four groups based on their structure (Figure 1): (i) 3-substituted phthalides, (ii) 3,3-disubstituted phthalides, (iii) non-3-substituted phthalides, and (iv) phthalide polymers (dimers and trimers) [2,7]. In several cases, the absolute configuration and bioactivity are unknown. There are over 200 naturally occurring phthalides isolated to date, with new compounds being discovered every year. Only in 2018, a dozen new phthalides were isolated from natural sources, testifying the prevalence of this class of compounds [7,8,9]. 

**Figure 2:**
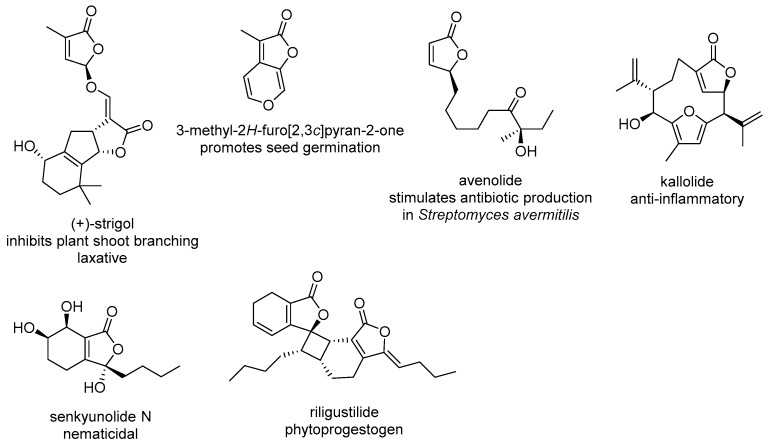
Representative examples of biologically active α,β-butenolides.

Structurally related to phthalides are α,β-butenolides, which lack a phenyl ring fused to the lactone. These compounds are also widespread in nature and show a variety of biological activities (Figure 2) [10]. For example, strigol stimulates the germination of parasitic plants [11] and inhibits plant shoot branching in rice [12]; it is also used as a laxative [13]. Avenolide is a fungal hormone that stimulates antibiotic production in *Streptomyces avermitilis* [14].

**Scheme 1:**
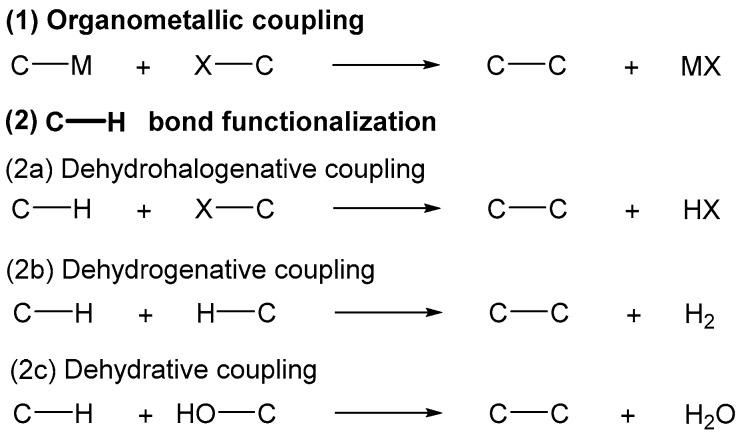
Classification of coupling reactions according to the functionalization level of reactants.

**Scheme 2:**
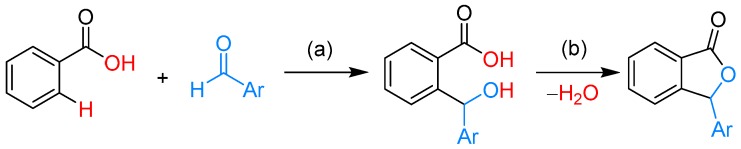
Cascade reaction of (**a**) Grignard-type reaction (via C—H bond activation) and (**b**) lactonization to afford 3-substituted phthalides.

Traditional approaches to the synthesis of phthalides and α,β-butenolides involve reactions that are either non-catalytic or multi-step [10,15]. An innovative approach is based on C—H bond functionalization, which has revolutionized organic synthesis allowing the use of simple, unactivated starting materials to make C—C bonds (Scheme 1, (2)) [16]. From an environmental viewpoint, the most useful C—H bond functionalization reactions are those in which both coupling partners are unactivated. Two types of reaction belong to this class: dehydrogenative coupling reactions, in which two C—H bonds are coupled to form a C—C bond with formal removal of a hydrogen molecule (Scheme 1, (2b)) [17], and dehydrative coupling reactions, in which a C—H and a C—OH bond react to form a C—C bond with elimination of a water molecule (Scheme 1, (2c)) [18]. These two classes of reactions offer a double advantage with respect to traditional methodologies: (1) they eliminate the need for the preparation and isolation of activated substrates prior to coupling (direct synthesis), and (2) they generate little or no waste (high atom economy). Such features comply with two essential principles of green chemistry [19], making dehydrogenative and dehydrative coupling reactions eco-sustainable.

The syntheses of phthalides and α,β-butenolides have recently been reviewed, but with little or no mention of the direct methods involving C—H bond functionalization [10,15]. This review fills the gap by surveying such methods. Each methodology is discussed in terms of scope and mechanism; however, reactions are classified by product structure rather than mechanism similarity, as the review is supposed to be mainly a guide for the synthetic chemist in the preparation of a specific class of compounds. Semi-direct methods requiring the pre-synthesis of starting materials in no more than three steps are included in the review, whereas methods based on dehydrohalogenative coupling reactions (Scheme 1, (2a)) are not discussed here.

## 2. Phthalides 

### 2.1. 3-Substituted Phthalides

#### 2.1.1. 3-Aryl Phthalides 

From Benzoic Acids and Aromatic Aldehydes [20]

One strategy to prepare 3-aryl phthalides by C—H bond functionalization is the annulative coupling of benzoic acids and aromatic aldehydes (Scheme 2). The reaction is a cascade process in which one aldehyde molecule is converted into a carbon nucleophile by *ortho*-C—H bond activation, then undergoes a Grignard-type addition to another aldehyde molecule (a). Finally, cyclization with elimination of a water molecule affords phthalide (b). 

**Scheme 3:**
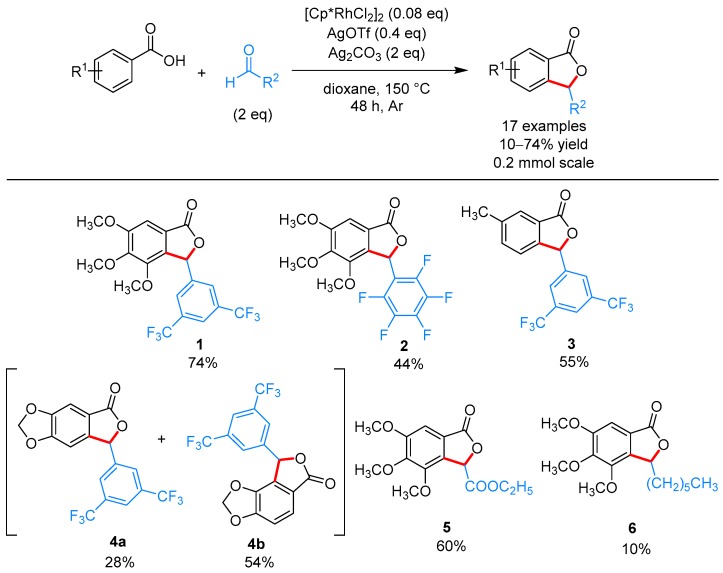
Rh-catalyzed annulative coupling of benzoic acid and aromatic aldehydes for the synthesis of 3-aryl phthalides.

The reaction is catalyzed by [Cp*RhCl_2_]_2_ in the presence of two silver additives (AgOTf and Ag_2_CO_3_) (Scheme 3). Both additives are necessary as no reaction takes place if either one is removed. Because the Grignard-type reaction takes place between a stabilized carbanion and an electrophile, the highest yields are obtained by a combination of electron-rich acids and electron-poor aldehydes (**1–3**). 

Aminobenzoic acids, despite being electron-rich, afford little or no product presumably due to the formation of an ammonium salt, which deactivates the benzene ring towards electrophilic C—H bond activation. Polyhydroxybenzoic acids are also unreactive, possibly due to deactivation resulting from coordination of rhodium to the OH groups. Ethyl oxoethanoate and heptanal afford the corresponding products **5** and **6** in 60 and 10% yield, respectively, suggesting that the methodology is suitable for multifunctional aliphatic aldehydes but not for simple aliphatic aldehydes. 3-Substituted benzoic acids, for which two cyclization positions are available, cyclize on the carbon atom at the *para* position with respect to the substituent with full regioselectivity, provided that the substituent plays no mesomeric effect (compare **3** with **4a** and **4b**).

**Scheme 4:**
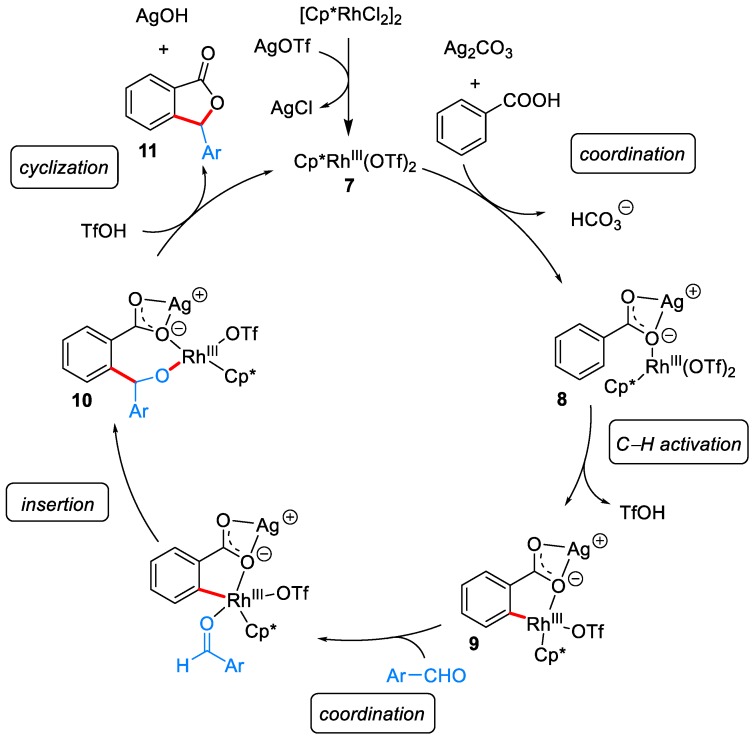
Proposed mechanism for the Rh-catalyzed coupling of benzoic acid and aromatic aldehydes.

**Scheme 5:**
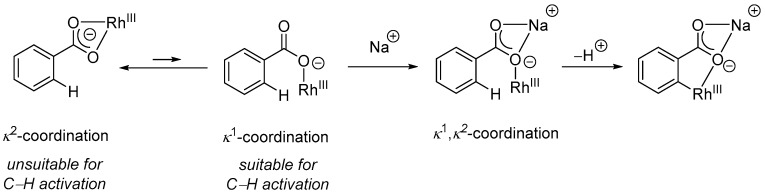
Effect of Na chelation on the coordination ability of the carboxylate group in benzoic acid.

According to the proposed mechanism (Scheme 4), AgOTf plays a critical role in the generation of the active species. This salt acts as a chloride trapping agent, removing chloride ions from [Cp*RhCl_2_]_2_ and generating Cp*Rh(OTf)_2_ (**7**), a highly electrophilic complex capable of undergoing C—H activation. Benzoic acid is deprotonated by carbonate ion and converted into benzoate, which coordinates to Rh(III) through one of the carboxylic oxygens giving rhodium benzoate **8**. Subsequent *ortho*-C—H bond activation via electrophilic aromatic substitution (S_E_Ar) forms rhodacycle **9**, releasing a proton at the same time. The aldehyde carbonyl coordinates to rhodium, then inserts into the C—Rh bond producing a seven-membered rhodium alkoxide (**10**). Protonation of the alkoxide favors an intramolecular nucleophilic substitution that provides the final product **11** and regenerates the active species **7**. The catalytic activity drops if Ag_2_CO_3_ is replaced with K_2_CO_3_, implying that silver ion is involved not only in the generation of the active species but also in the catalytic cycle. The catalytic effect of Ag^+^ ion can be rationalized by the model of weak coordination mode proposed for Na^+^ and carboxylate ions (Scheme 5) [21]. In the specific case, Ag(I) binds to the carboxylate group in a *κ*^2^ coordination mode, inducing Rh(III) to coordinate with an oxygen lone pair in a *κ*^1^ fashion. This structure allows the rhodium center to approach the *ortho*-position of the benzene ring and to trigger C—H activation. In the absence of silver ions, Rh(III) would preferentially coordinate to the carboxylate group in a *k*^2^ fashion, which is not suitable for C—H activation.

From Two Aromatic Aldehydes [22]

**Scheme 6:**
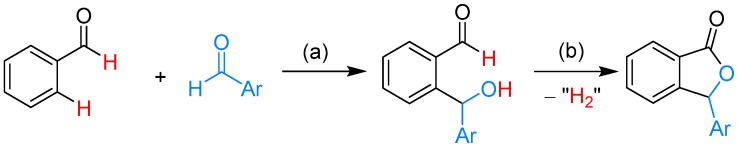
Cascade reaction of (**a**) Grignard-type reaction (via C—H bond activation) and (**b**) dehydrogenative cyclization to afford 3-substituted phthalides.

An alternative direct method for the synthesis of 3-aryl phthalides is the coupling of two aromatic aldehydes, with one supplying the benzoyl moiety and the other the remaining fragment of phthalide (Scheme 6). 

**Scheme 7:**
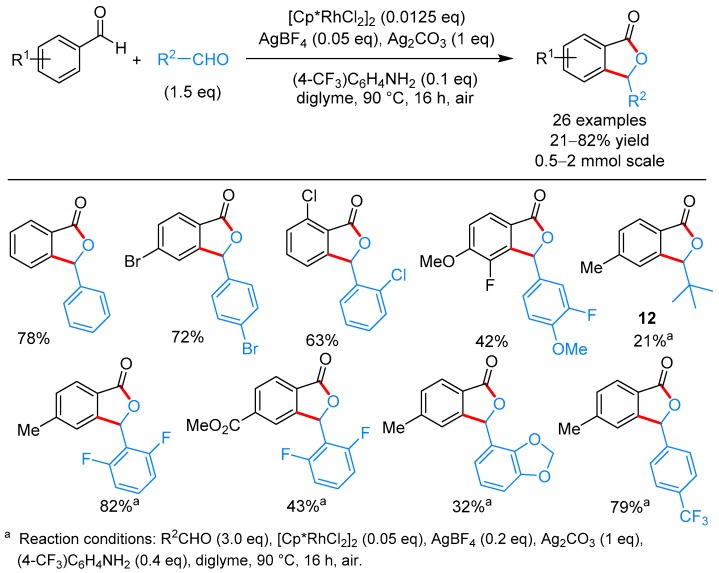
Rh-catalyzed annulative coupling of benzaldehydes for the synthesis of 3-substituted phthalides.

The reaction is analogous to the one reported above and runs under similar conditions ([Cp*RhCl_2_]_2_ catalyst and stoichiometric amounts of Ag_2_CO_3_) (Scheme 7); however, Ag_2_CO_3_ is working as an oxidant here, as the overall reaction is a dehydrogenation. The reaction also requires an amine co-catalyst, which likely acts by activating the carbonyl aldehyde in the form of an imine. Consistently with this hypothesis, the electron-poor 4-trifluoromethylaniline is much a better co-catalyst than unsubstituted aniline or electron-rich *N*-methylaniline (69, 28, and 10% yield for the homocoupling reaction of benzaldehyde at 130 °C in 1.5 h, respectively). The use of a metal oxidant in the place of molecular oxygen is of paramount importance to avoid the oxidation of benzaldehyde to benzoic acid. For the same reason, the reaction needs to be performed under nitrogen. Both homo- and hetero-coupling of aromatic aldehydes can be performed under this methodology, with electron-withdrawing substituents being tolerated on both rings. However, heterocoupling requires larger amounts of catalyst and additives as well as a larger excess of the aldehyde being used as the electrophile (R^2^CHO); the by-product from the homocoupling of excess aldehyde is also obtained in this case. The introduction of aliphatic substituents in position 3 of the lactone ring is possible but not useful for synthetic purposes due to low (<25%) yields (e.g., **12**).

**Scheme 8:**
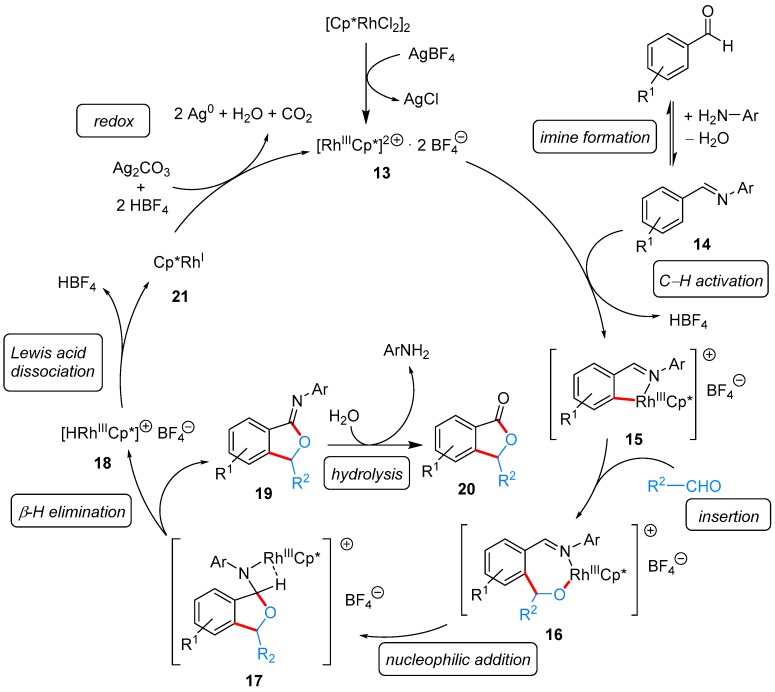
Proposed mechanism for the Rh-catalyzed annulative coupling of benzaldehydes.

The active species in the catalytic cycle is likely a dicationic Rh(III) complex (**13**) generated in situ from [Cp*RhCl_2_]_2_ and AgBF_4_ (Scheme 8). The rhodium species activates the *ortho* C—H bond of the imine derivative **14**, which is also generated in situ by aldehyde imination. The C=O group of another aldehyde molecule inserts into the Rh—C bond of **15** to form the rhodacycle **16**. The alkoxy group of **16** undergoes intramolecular nucleophilic addition on imine with formation of spiro-metallacycle **17**. β-Hydride elimination in **17** affords the Rh(III) hydride **18** and the imine intermediate **19**, which then hydrolyzes to form phthalide **20**. The Rh(III)—H species loses a proton to generate a Rh(I) complex (**21**), which is oxidized by Ag_2_CO_3_ to regenerate the active species (**13**). 

From *N*-Substituted Benzimidates and Aromatic Aldehydes [23]

**Scheme 9:**
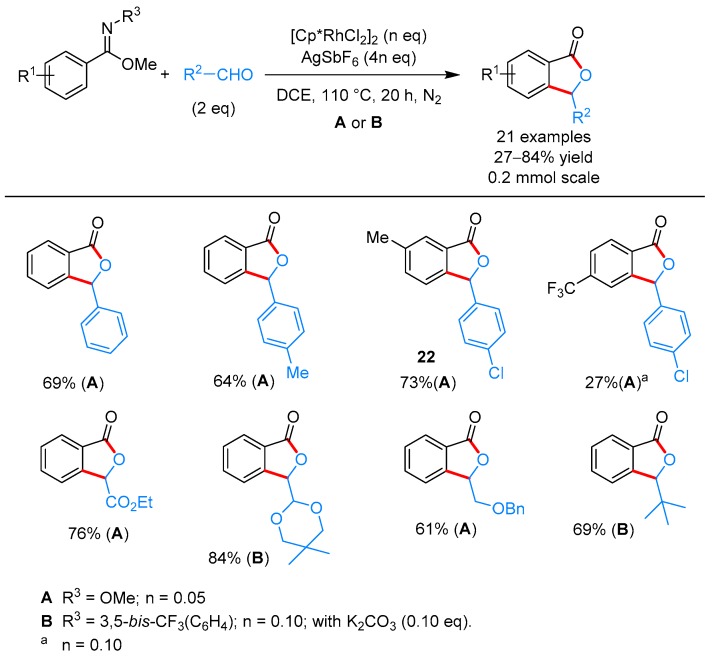
Rh-catalyzed annulative coupling of methyl *N*-substituted benzymidates and aldehydes for the synthesis of 3-substituted phthalides.

**Scheme 10:**
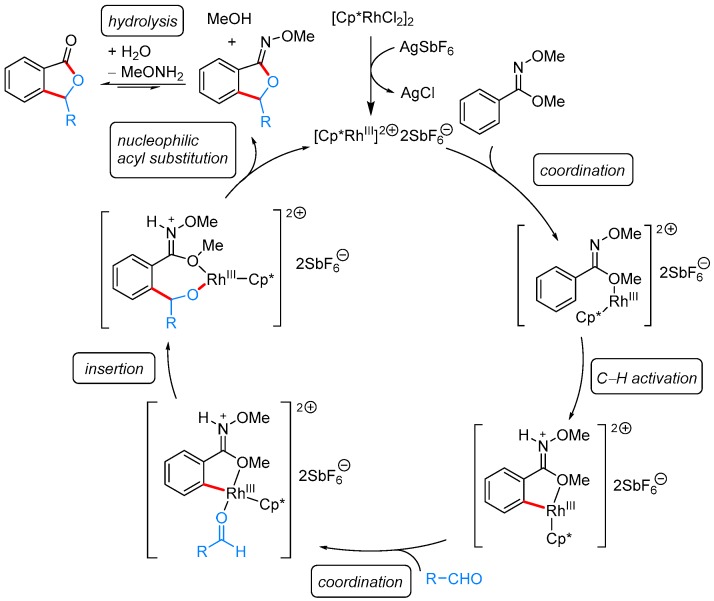
Possible mechanism for the Rh-catalyzed annulative coupling of methyl *N*-methoxybenzymidate and aldehydes.

**Scheme 11:**
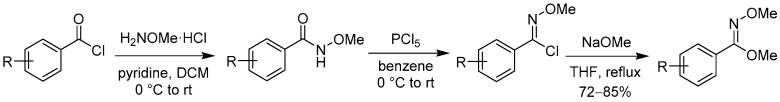
Multi-step synthesis of methyl *N*-methoxybenzimidates from benzoyl chlorides.

**Scheme 12:**
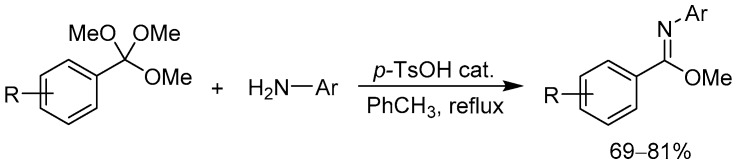
Synthesis of methyl *N*-arylbenzimidates from trimethyl orthobenzoates.

A strategy analogous to the one described above involves the use of *N*-substituted benzimidates in the place of benzoic acids (Scheme 9). The imidate group acts in the same way as a carboxylic group, promoting *ortho*-C—H activation followed by aldehyde insertion and nucleophilic acylic substitution of the resulting alkoxide onto the imidate (Scheme 10). Final hydrolysis of imidate releases the phthalide. Consistently with this mechanism, the effect of substituent is the same as that observed for the coupling of benzoic acids and aldehydes. The substituent on nitrogen plays a key role in the reaction: the *N*-methoxy group is suitable for coupling of aromatic aldehydes, whereas *N*-aryl groups—particularly the electron-deficient 3,5-*bis*-CF_3_(C_6_H_4_) group—is mostly indicated for aliphatic aldehydes. The reason for such preference remains unclear. This method has a wider scope than those described above, as it tolerates both aromatic and aliphatic aldehydes (simple and functionalized). Additionally, the steric hindrance of substituent on nitrogen ensures full control of regioselectivity, with *meta*-methyl benzimidate affording the product resulting from C—H activation at the less hindered site only (**22**). Unfortunately, the large scope and high regioselectivity come at the expense of simplicity: this method is not direct because it requires the pre-synthesis of benzimidates in three steps (Scheme 11 and Scheme 12). One precaution to be adopted when using aliphatic aldehydes and *N*-methoxybenzimidate is the addition of K_2_CO_3_ (0.5 eq) to minimize the acid-catalyzed aldol condensation.

#### 2.1.2. 3-Alkyl Phthalides 

##### From Benzoic Acids and Electrophilic Alkenes

**Scheme 13:**
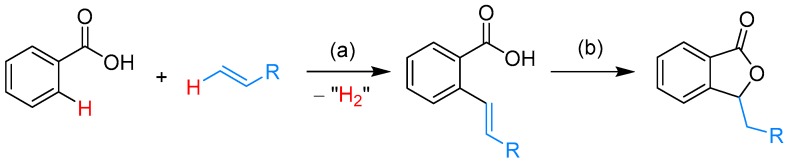
Cascade reaction of (**a**) Heck-type dehydrogenative alkenylation and (**b**) Michael-type addition to afford 3-substituted phthalides.

A major strategy for the synthesis of 3-alkyl phthalides is the coupling of benzoic acids and electron-poor alkenes (Scheme 13). This domino reaction proceeds through an Heck-type *ortho*-alkenylation (a), followed by an intramolecular Michael-type addition (b). The alkenylation, being a dehydrogenative coupling, requires the use of an oxidant. Unlike arylation, alkenylation poses a chemoselectivity issue because it may occur at both *ortho*-positions, affording a mixture of 3-substituted and 3,7-disubstituted products. Various methodologies have been developed to perform this domino reaction, but only a few are fully chemoselective.

Ru^II^/Cu^II^ System [24,25,26]

**Scheme 14:**
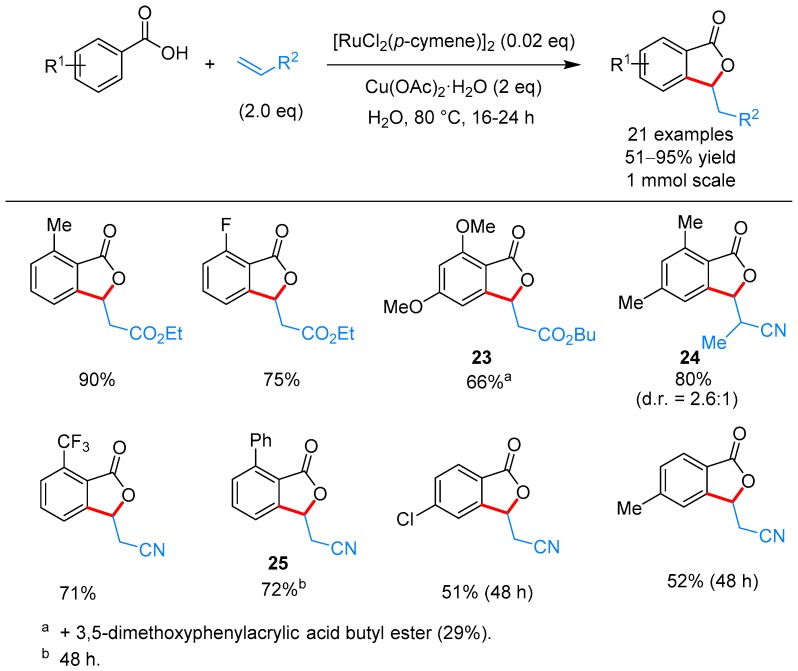
Rh-catalyzed coupling of benzoic acids and electrophilic alkenes in water for the synthesis of 3-substituted phthalides.

**Scheme 15:**
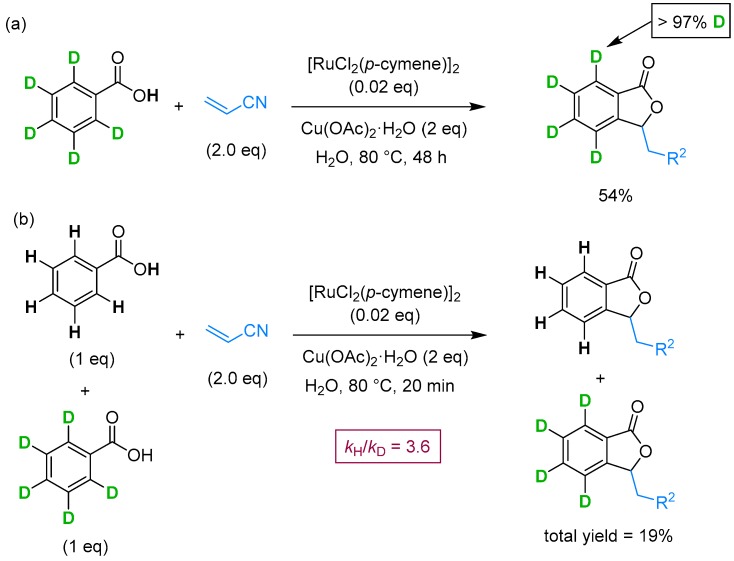
Deuterium-labelling experiments for the Rh-catalyzed coupling of benzoic acids and electrophilic alkenes in water.

One fully chemoselective method for the synthesis of 3-alkyl phthalides is the coupling of benzoic acids and electrophilic alkenes in the presence of [RuCl_2_(*p*-cymene)]_2_ as catalyst and Cu(OAc)_2_·H_2_O as oxidant in water [24] (Scheme 14). The protocol tolerates both electron-withdrawing and electron-donating groups on the phenyl ring, but is restricted to acrylonitriles and acrylic esters as the alkenes. Only unsubstituted and *ortho*-substituted benzoic acids have been used with this methodology, so the reaction regioselectivity is unknown. A single example (**24**) of diastereoselective reaction has been reported in the presence of *geminal*-disubstituted alkenes, though with modest diastereoselectivity (d.e. <50%). Besides chemoselectivity, the main advantages of this methodology are the use of a cheap catalyst and a safe solvent. Reaction of acrylonitrile and PhCOOH-*d*_5_ highlights almost no H/D exchange, showing that *ortho*-C—H bond cleavage is irreversible (Scheme 15a). Furthermore, an intermolecular competition experiment provides a kinetic isotope effect (KIE) of *k*_H_/*k*_D_ = 3.6, pointing to C—H activation as the rate-limiting step (Scheme 15b). The addition of a radical scavenger (TEMPO) does not inhibit the catalytic activity, ruling out the possibility of a SET mechanism. On the contrary, a slight increase in yield (from 90 to 95%) is obtained when TEMPO is used as an additive in the reaction of *o*-toluic acid and ethyl acrylate, suggesting a general strategy to improve the reaction performance. In one case (**23**) the reaction affords the lactone along with the product of an alkenylation/decarboxylation cascade reaction. This observation provides evidence for a mechanism proceeding via *ortho*-alkenylation.

**Scheme 16:**
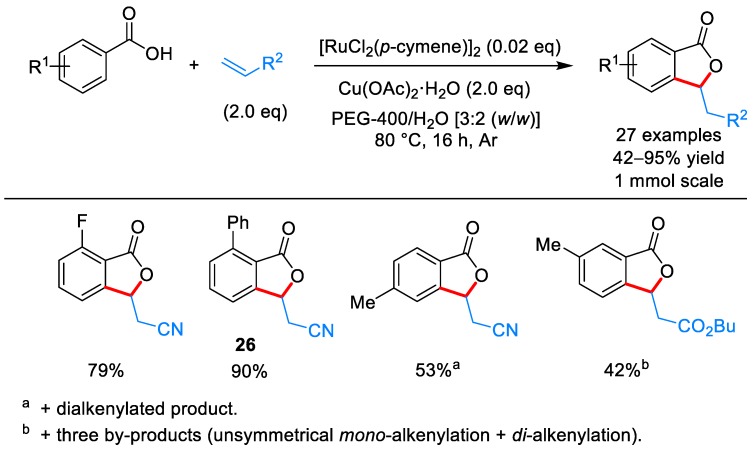
Ru-catalyzed annulative coupling of benzoic acids and electrophilic alkenes in PEG-400/water for the synthesis of 3-substituted phthalides.

If the coupling of benzoic acids and electrophilic alkenes is performed in a mixture of polyethylene glycol 400 (PEG-400) and water (3:2 [*w*/*w*]) instead of pure water, the catalyst can be recycled and reused up to six times with no loss of catalytic activity (Scheme 16) [25]. This improvement is made possible by a change in work-up. The reaction in pure water [24] is worked up with saturated aq. NH_4_Cl/NH_3_ (1:1), which removes not only Cu(II) but also the ruthenium catalyst. In contrast, PEG-400/H_2_O mixture strongly holds both metal complexes, presumably due to coordination of polymer oxygens to metal; as a consequence, no work-up is necessary and the product can be easily recovered by simple extraction with petroleum ether. In some cases, a significant (>10%) increase in reaction yield is obtained on switching solvent from pure water to PEG-400/H_2_O (compare the yields of **25** in Scheme 14 and **26** in Scheme 16). Unfortunately, the use of PEG-400 also determines a loss of chemoselectivity: 4-substituted benzoic acids afford a mixture of *mono*- and *di*-alkenylated products, whereas 3-substituted benzoic acids provide four products (two *mono*-alkenylated and two *di*-alkenylated). Thus, the presence of an *ortho*-substituent is necessary to avoid disubstitution. The alkene scope is still limited to vinyl acrylates and acrylonitrile; acrylamides are unreactive under these conditions.

**Scheme 17:**
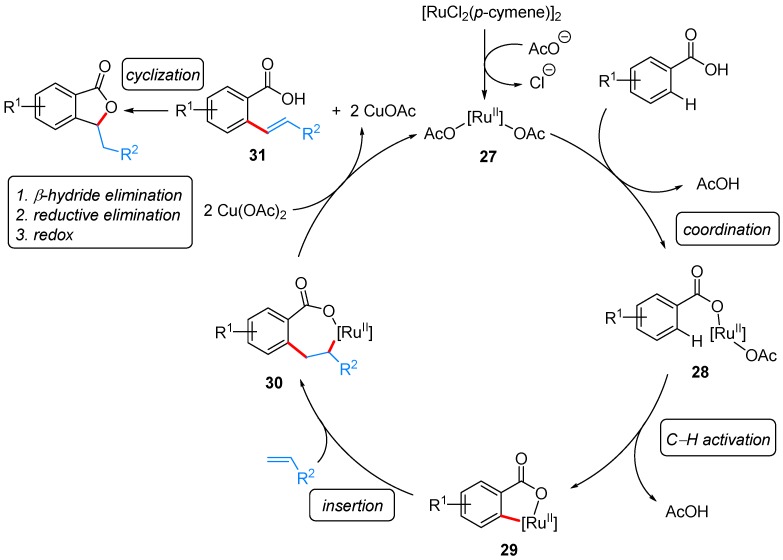
Proposed mechanism for the Ru-catalyzed annulative coupling of benzoic acids and alkenes in aqueous systems.

According to the proposed mechanism (Scheme 17), Cu(OAc)_2_·H_2_O serves as a source of both Cu^2+^ (oxidant) and AcO^−^, which is necessary to generate the active species Ru(OAc)_2_ (**27**). Coordination of the carboxyl oxygen of benzoic acid to **27** with liberation of AcOH gives ruthenium benzoate **28**. Then, *ortho*-metalation of **28** affords metallacycle **29**. Alkene insertion into the Ru—C bond affords the seven-membered ruthenacycle **30**. **30** undergoes β-hydride elimination followed by reductive elimination to produce the *ortho*-alkenylated intermediate **31**, which cyclizes via an intramolecular Michael-type addition to generate the product. In principle, cyclization may be catalyzed by Cu(II) or AcOH, or both. Finally, Ru(0) is reoxidized to Ru(II) by Cu(II), with regeneration of **27**. 

**Scheme 18:**
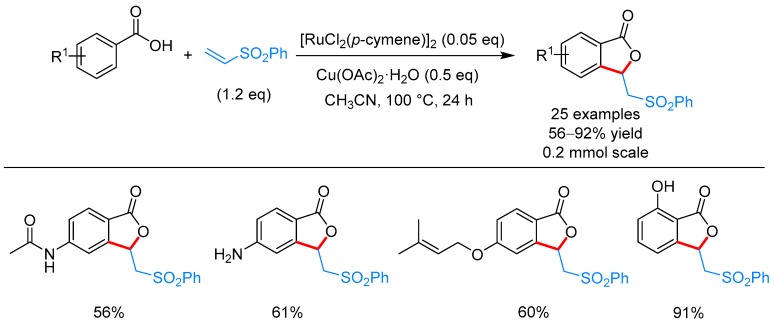
Ru-catalyzed annulative coupling of benzoic acids and electrophilic alkenes for the synthesis of 3-substituted phthalides in the presence of Cu(II).

The scope of the Ru-catalyzed coupling can be extended to aryl vinyl sulfones using acetonitrile as the solvent (Scheme 18) [26]. It is unclear whether this reaction can also be performed in water because this solvent has not been screened by authors. 

Ru^II^/O_2_ System [27,28]

**Scheme 19:**
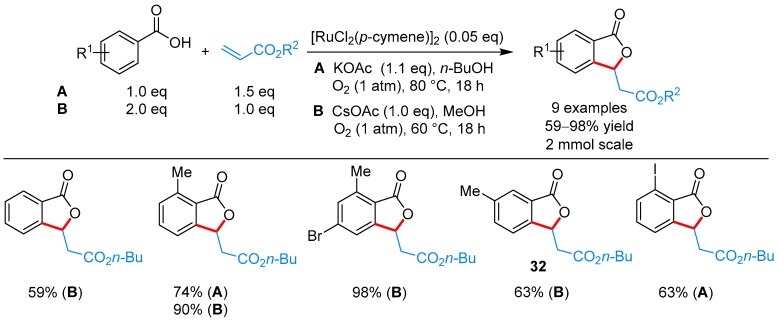
Ru-catalyzed annulative coupling of benzoic acids and electrophilic alkenes for the synthesis of 3-substituted phthalides in alcohols the presence of O_2_.

**Scheme 20:**
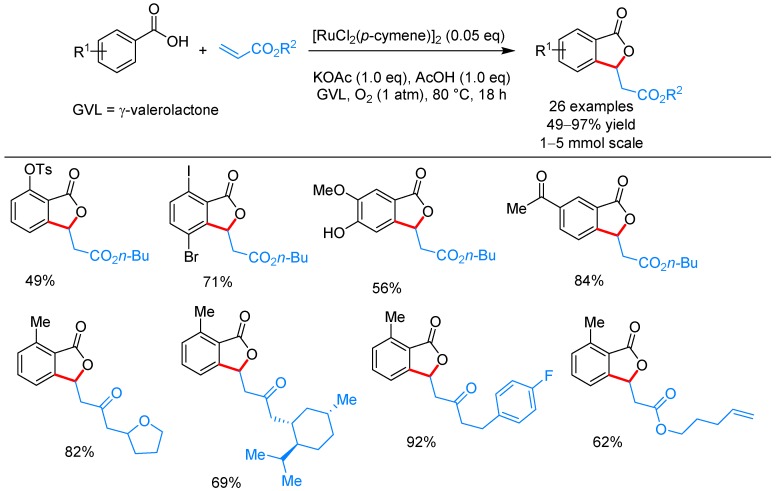
Ru-catalyzed annulative coupling of benzoic acids and electrophilic alkenes for the synthesis of 3-substituted phthalides in GVL in the presence of O_2_.

The Ru-catalyzed annulative coupling of benzoic acids and alkenes can be performed using O_2_ as the sole oxidant (Scheme 19 and Scheme 20). This approach is much more green than other strategies involving metal oxidants, because the reaction of O_2_ and “H_2_” issued from dehydrogenative coupling produces water as the only by-product. The catalyst has been defined a ruthenium oxidase by analogy with biological oxidases. Two solvent types can be used with this catalytic system: alcohols (MeOH or *n*-BuOH, Scheme 19) [27] and GVL (γ-valerolactone, Scheme 20) [28]. Both can be considered sustainable solvents as they are derived from biomass. With respect to alcohols, GVL offers the advantage of avoiding undesirable transesterification reactions and solvent oxidation. Additionally, GVL is less flammable and safer than *n*-BuOH due to its higher flash point (96 °C *vs* 35 °C). The hazard of fire associated with the use of molecular oxygen in the presence of flammable organic solvents can be avoided completely using H_2_O_2_ as the terminal oxidant. The change in oxidant from Cu(OAc)_2_·H_2_O to O_2_ does not modify the reaction chemo- and regio-selectivity: total chemoselectivity is observed, with 3-substituted phthalides being obtained exclusively; no dialkenylation occurs. Full regioselectivity is also observed for *meta*-substituted benzoic acids (e.g., **32**). The reaction in GVL has been scaled up to the gram scale with a yield increase with respect to the mmol scale.

**Scheme 21:**
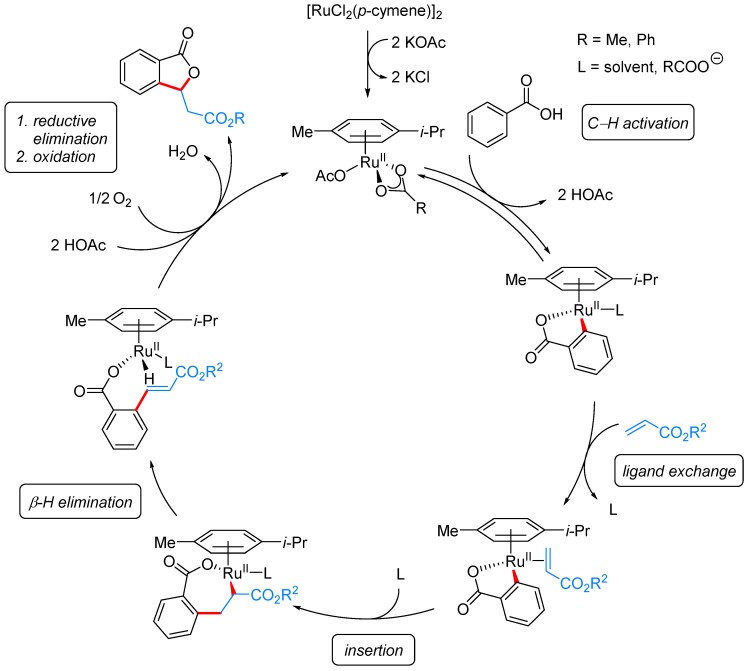
Mechanism for the Ru-catalyzed annulative coupling of benzoic acids and alkenes in the presence of O_2_.

The proposed catalytic cycle (Scheme 21) is analogous to that proposed for the Ru^II^/Cu^II^ system (Scheme 17). The weak coordinating ability of carboxylate group to Ru^II^ is deemed crucial for the ligand exchange reaction between acetate and benzoate at the beginning of the catalytic cycle.

Rh^III^/Cl^III^ System [29]

**Scheme 22:**
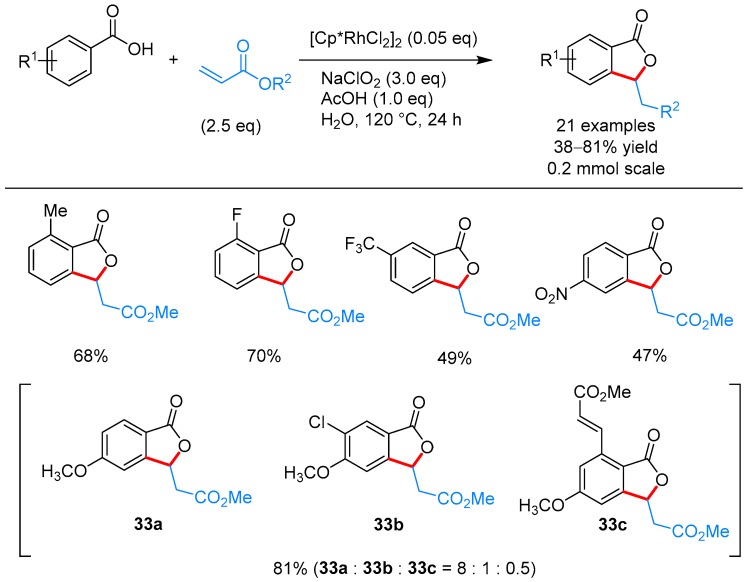
Rh-catalyzed annulative coupling of benzoic acids and electrophilic alkenes for the synthesis of 3-substituted phthalides in the presence of Cl^III^.

**Scheme 23:**
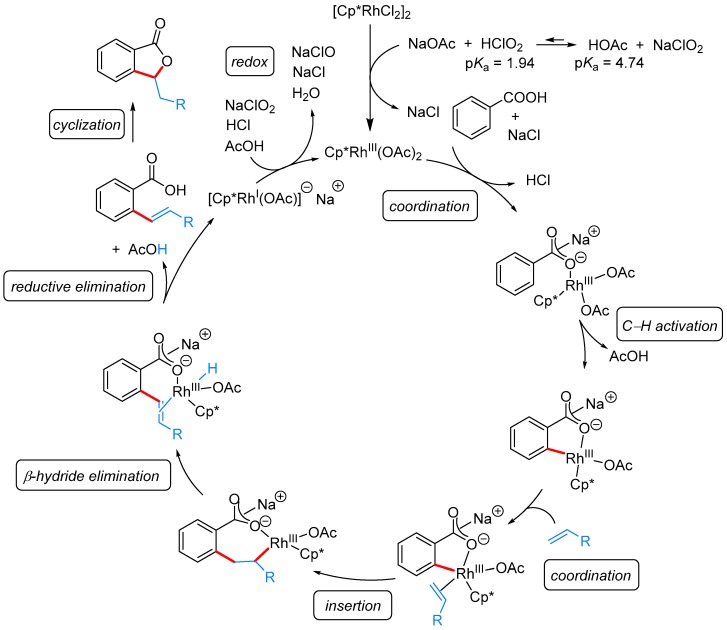
Proposed mechanism for the Rh-catalyzed annulative coupling of benzoic acids and electrophilic alkenes in the presence of Cl^III^.

Another method to achieve the chemoselective coupling of benzoic acids and alkenes involves the use of [Cp*RhCl_2_]_2_ as the catalyst and NaClO_2_ as the oxidant in H_2_O/AcOH (Scheme 22). The nature of the acid additive is crucial to the reaction outcome because the yield is halved when acetic acid is replaced with pivalic acid or trifluoroacetic acid. Both acetic acid and pivalic acid perform better than CF_3_COOH, suggesting that a coordinating anion is needed to stabilize one or more intermediates in the catalytic cycle. Consistently with this hypothesis, pivalic acid provides a lower yield than acetic acid due to its steric hindrance and lower coordination ability. This method is similar to the one based on ruthenium catalysis in terms of chemoselectivity, scope, and eco-friendliness. Chemoselectivity is attributed by authors to the specific oxidizing ability of sodium chlorite in acidic solution, although it may be simply due to the effect of a protic solvent, analogously to the ruthenium catalysis in water. NaClO_2_ can be considered a relatively safe oxidant, as it only produces NaCl as waste; however, it also introduces a source of chlorine that may result into chlorination of the phenyl ring. For instance, the reaction of 4-methoxybenzoic acid affords the 3-substituted phthalide **33b** chlorinated in the *meta* position, together with 3-substituted phthalide **33a**; a small amount of 3,7-disubstituted product (**33c**) is also recovered. This is the only non-chemoselective example reported by authors. Although the effect of oxidant cation has not been investigated, the role of Na^+^ in enhancing the C—H activation rate by weak coordination to the carboxylate group can be envisaged (Scheme 23) [21]. 

Rh^I^/Cu^II^ System [30]

**Scheme 24:**
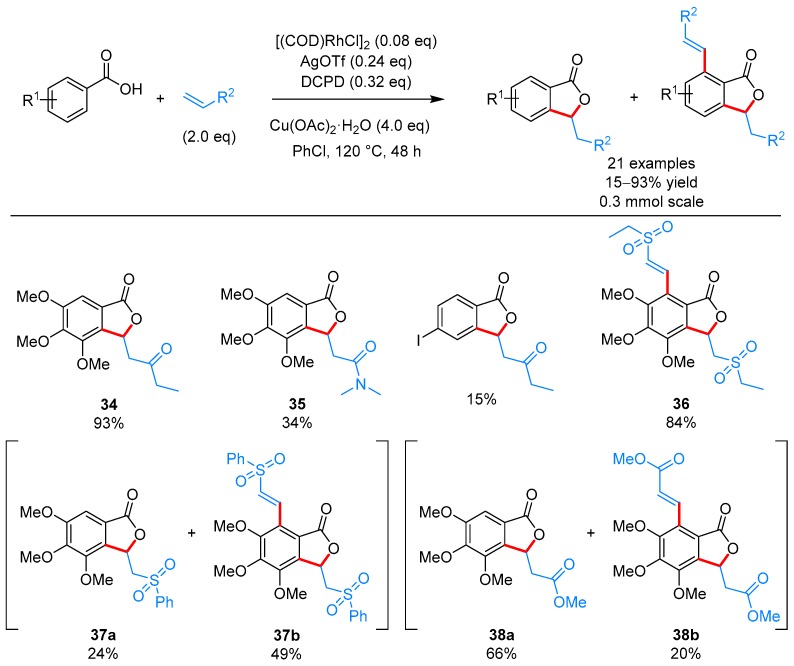
Rh-catalyzed annulative coupling of benzoic acids and electrophilic alkenes for the synthesis of 3-substituted phthalides in the presence of Cu(II).

**Scheme 25:**
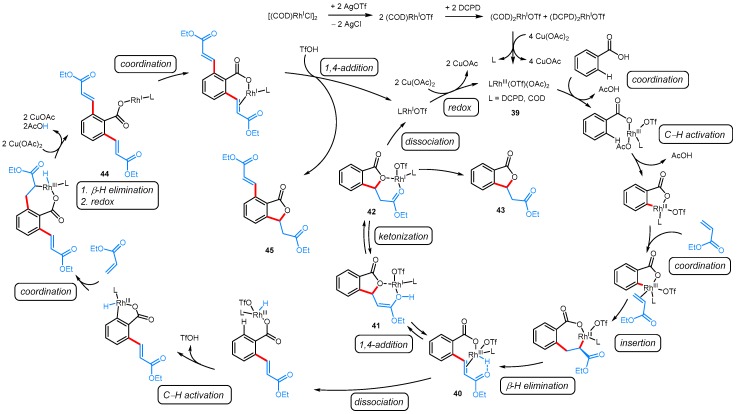
Proposed mechanism for the Rh-catalyzed annulative coupling of benzoic acids and electrophilic alkenes in the presence of Cu(II).

An additional method for the synthesis of 3-alkyl phthalides from benzoic acids and alkenes uses [(COD)RhCl]_2_ as the catalyst, Cu(OAc)_2_·H_2_O as the oxidant, and dicyclopentadiene (DCPD) as the additive (Scheme 24). Under such conditions, chemoselectivity depends on alkene type: α,β-unsaturated ketones and amides afford 3-substituted phthalides exclusively (**34** and **35**); α,β-unsaturated esters and aromatic vinyl sulfones provide a mixture of 3-substituted and 3,7-disubstituted products (**37a** + **37b** and **38a** + **38b**); ethyl vinyl sulfone only affords a 3,7-disubstituted product (**36**). Product **36** is the only example of 3,7-disubstituted phthalide obtained chemoselectively by a direct method. Catalyst and additive are also crucial for chemoselectivity, because other metal complexes such as (Cp*RhCl_2_)_2_ and [Cp*Rh(MeCN)_3_](SbF_6_)_2_, or additives like Ph_4_CpH_2_ and Me_5_CpH lead to a mixture of 3-substituted and 3,7-disubstituted products (Table 1). ^1^H-NMR and X-ray crystallography have shown that DCPD undergoes a ligand exchange reaction with [(COD)RhCl]_2_ generating two homoleptic rhodium complexes [(COD)_2_RhOTf and (DCPD)_2_RhOTf], which are both catalytically active (Scheme 25). Oxidation of Rh(I) to Rh(III) in situ generates the active species **39**. *Di*-substituted product **45** is not formed from the monosubstituted product **42**, because treatment of **42** with 1 eq of alkene does not lead to **45** under the catalytic conditions. **42** and **45** come from a common intermediate (**40**), which is formed by a classical metal-catalyzed *ortho*-alkenylation of benzoic acid. **40** can follow two pathways. In the first one, it undergoes an intramolecular Michael addition generating enol **41**, which tautomerizes to the thermodynamically more stable keto form **42**; final dissociation of rhodium affords monosubstituted product **43**. In the second one, rhodium dissociates from the C=C bond in **40**, then catalyzes the coupling of a second molecule of alkene at the other *ortho* position giving intermediate **44**; Michael addition of the carboxylate group to either vinylic group of **44** results into disubstituted product **45**. In both pathways, Rh(I) is reoxidized to Rh(III) by Cu(II) with regeneration of the active species. The observed chemoselectivity, i.e., the preferential formation of **43** or **45**, is likely related to the rate of ketonization of enol **41**, which in turn depends on the nature of the electron-withdrawing group (EWG) on alkene. If ketonization of **41** is slow (EWG = CO_2_Me, SO_2_Ph, SO_2_Et), **41** reverses to **40** and undergoes a second alkenylation before ring formation, affording **44** and eventually **45**. If ketonization of **41** is fast (EWG = COMe, CONMe_2_), **41** is trapped into **42** as soon as it is formed, so the retro-Michael addition cannot take place; then, **42** is converted into **43**. This mechanism may also explain why chemoselectivity is metal- and ligand-dependent: metal and ligands, similarly to substrate, can affect the stability of intermediates **40** and **41** and hence the relative rates of ketonization and conjugate addition. 

Rh^III^/O_2_ System [31]

**Scheme 26:**
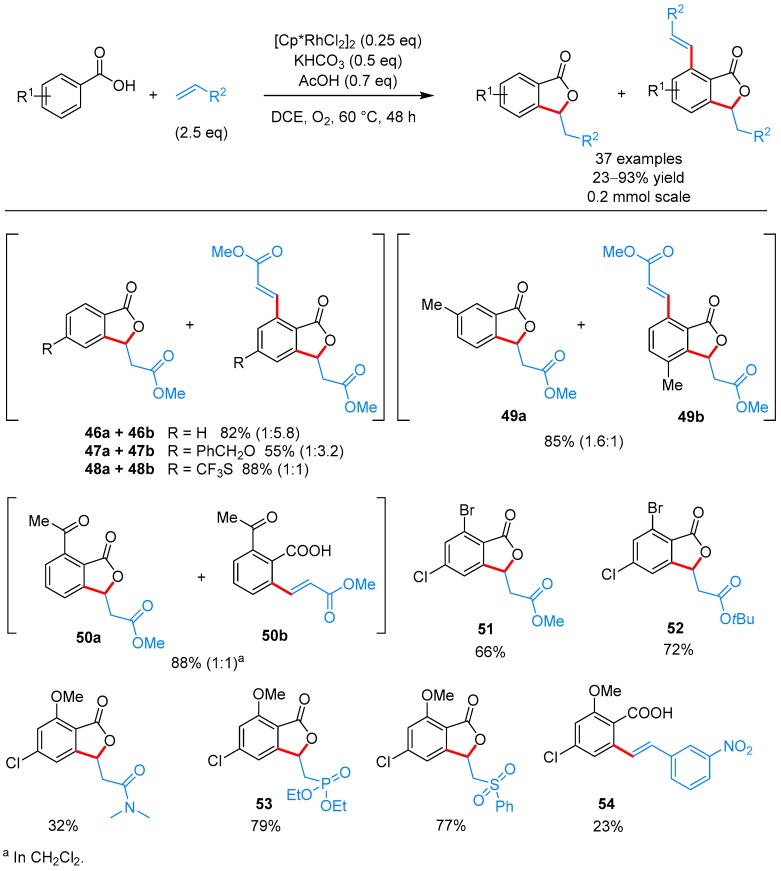
Rh-catalyzed annulative coupling of benzoic acids and electrophilic alkenes for the synthesis of 3-substituted phthalides in the presence of O_2_.

Similarly to Ru(II), Rh(III) can catalyze the annulative coupling of benzoic acids and alkenes under an oxygen atmosphere without any external oxidant (Scheme 26). However, in this case the reaction is not fully chemoselective, and a mixture of *mono*- and *di*-substituted products is obtained, unless the benzene ring is *ortho*-substituted. The major product depends on the nature and position of substituents on the phenyl ring: *m*-substituted benzoic acids mainly afford 3-substituted phthalides (**49a**); unsubstituted and *p*-substituted benzoic acids preferentially give 3,7-disubstituted phthalides (**46b** and **47b**). The only exception to this pattern is *p*-CF_3_S(C_6_H_4_)COOH, for which no selectivity is observed (**48a**:**48b** = 1:1). Remarkably, the reaction of *m*-toluic acid and methyl acrylate is fully regioselective, affording only two out of four possible products: monosubstituted phthalide **49a**, in which cyclization took place at the *para* position with respect to the methyl group, and disubstituted phthalide **49b**, in which the lactone ring is adjacent to methyl. This result suggests a difference in cyclization rate between the two monoalkenylated intermediates: when alkenylation takes place at the *para* position with respect to methyl, the vinyl group senses only a weak inductive effect from the substituent, therefore is reactive and cyclizes immediately yielding **49a**; conversely, the intermediate with the vinyl group adjacent to methyl is less prone to cyclization due to the stronger electron-donating effect of the methyl group, so the carboxylic group has time to rotate and promote a second alkenylation at the other free *ortho*-position before ring closing. It is unclear, though, why cyclization in **49b** occurs at the most hindered position.

This direct method, although not fully chemoselective, is useful as it allows the preparation of 3-substituted dihalogenated phthalides (**51** and **52**), which cannot be obtained by other direct methods. Remarkably, diethyl vinylphosphonate can be used as the coupling partner (**53**). With 1-nitro-3-vinylstyrene the reaction stops at the alkenylation step (**54**) and proceeds in low (23%) yield, possibly due to styrene homocoupling under acidic conditions [32]. The reaction of *ortho*-acetyl benzoic acid affords a mixture of lactone **50a** and uncyclized product **50b** in 1:1 ratio, probably due to the formation of an intramolecular hydrogen bond between the carboxyl and acetyl groups which hampers cyclization. 

**Scheme 27:**
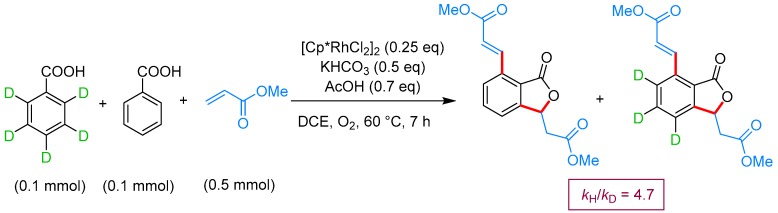
Deuterium-labelling experiments for the Rh-catayzed coupling of benzoic acids and electrophilic alkenes in the presence of O_2_.

An intermolecular competition experiment between PhCOOH and PhCOOH-*d*_5_ highlighted a KIE (*k*_H_/*k*_D_) ≫ 1, indicative of a rate-limiting C—H activation (Scheme 27).

Pd^II^/Cu^II^ System [33]

**Scheme 28:**
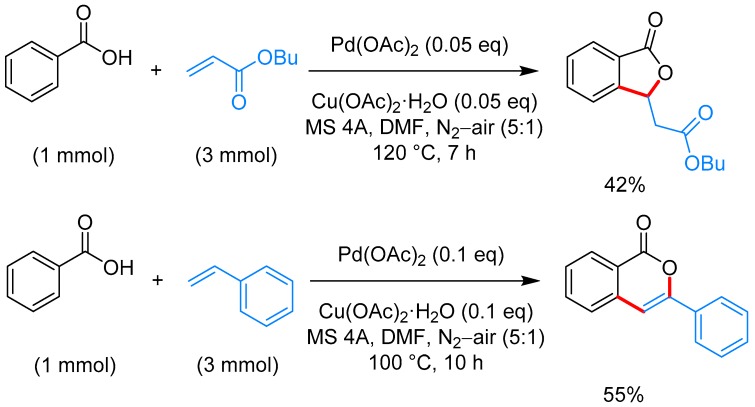
Pd-catalyzed annulative coupling of benzoic acid and alkenes for the synthesis of 3-substituted phthalides or 3-isocoumarins.

The first example of direct synthesis of 3-alkyl phthalides was reported over 40 years ago using a Pd(II) catalyst (Scheme 28). Reaction of benzoic acid and *n*-butyl acrylate in the presence of catalytic amounts of Pd(OAc)_2_ and Cu(OAc)_2_·H_2_O produces the corresponding 3-alkyl phthalide. As the copper salt is used in substoichiometric amount and the reaction is performed under a large volume (900 mL) of partially oxygenated atmosphere (N_2_/air = 5:1), oxygen is reasonably the terminal oxidant. If *n*-butyl acrylate is replaced with styrene, the reaction affords an isocoumarin instead of a phthalide, showing that the reaction chemoselectivity depends on the alkene type. This methodology, though old and limited to few examples, is important as it set the basis for further research in subsequent years. 

Rh^III^/Cu^II^ System [34]

**Scheme 29:**
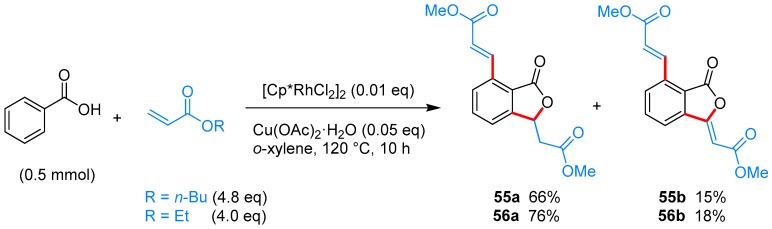
Rh-catalyzed annulative coupling of benzoic acid and electrophilic alkenes for the synthesis of 3-alkyl- and 3-ylidene phthalides.

If Pd(OAc)_2_ is replaced with [Cp*RhCl_2_]_2_ and a low-polarity solvent such as *o*-xylene is used in the place of DMF, the reaction of alkyl acrylates and benzoic acid affords no monosubstituted phthalide. Two disubstituted products are isolated instead: 3-alkyl-7-vinylphthalide (**55a** or **56a**) and 3-alkylidene-7-vinylphthalide (**55b** or **56b**) (Scheme 29). This outcome is different from that of the palladium-catalyzed reaction seen above, in which cyclization selectively occurs after the first vinylation, and confirms the strong dependence of chemoselectivity from the reaction conditions. 

From Benzamides and Electrophilic Alkenes [35]

**Scheme 30:**
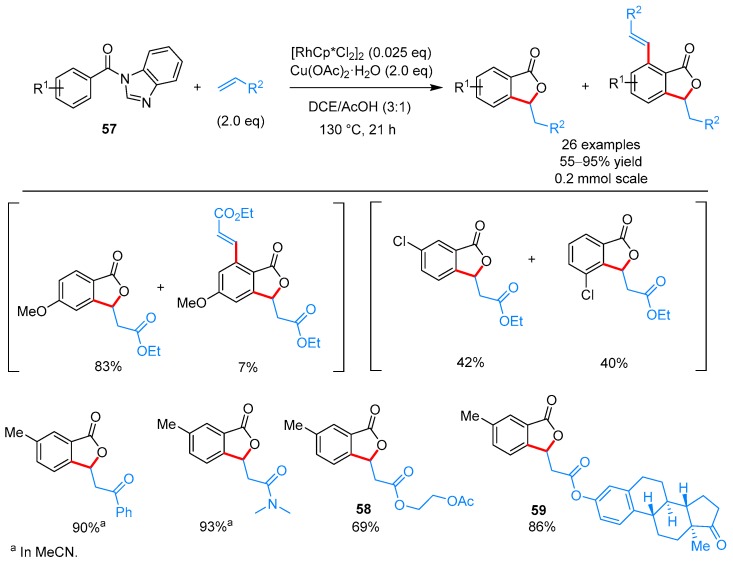
Rh-catalyzed annulative coupling of *N*-benzoyl benzimidazole and electrophilic alkenes for the synthesis of 3-substituted phthalides.

**Scheme 31:**
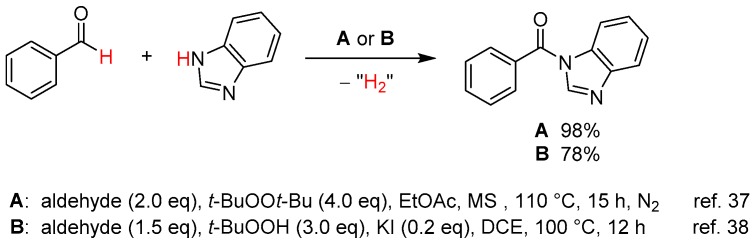
Synthesis of benzoyl benzimidazole by dehydrogenative coupling of benzaldehyde and benzimidazole.

**Scheme 32:**
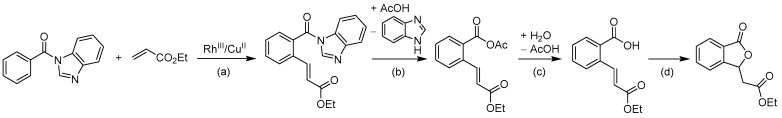
Mechanism outline for the Rh-catalyzed annulative coupling of benzoyl benzimidazole and ethyl acrylate: (**a**) *ortho*-alkenylation, (**b**) acetylation, (**c**) hydrolysis, and (**d**) cyclization.

Benzamides can be used in the place of benzoic acids as coupling partners in dehydrogenative coupling reactions to form phthalides, provided that a tertiary benzamide is used. The best results are obtained with benzamide derivative **57** containing a benzimidazole group (Scheme 30). All reactions provide phthalides as major products. This outcome is surprisingly different from the reaction of benzimides and internal alkynes, which mainly affords indenones [36]. An acid additive (AcOH) and a low-polarity solvent (DCE) are necessary to maximize yield and selectivity. Remarkably, most *meta*-substituted benzamides are only coupled at the less hindered position, possibly due to the large size of the benzimidazole directing group. The reaction has quite a large scope, being compatible with both electron-withdrawing and electron-donating substituents on the benzene ring of benzamide. 2-Hydroxyethyl acrylate (a polyfunctional alkene containing a primary hydroxyl group) can participate in the coupling reaction, although the hydroxyl group is acetylated during reaction (**58**). Estrone-containing acrylate can also be coupled with benzamide (**59**). The main drawback of this methodology is that benzoyl benzimidazole is not commercially available and needs to be prepared; however, it can be obtained in a single step by dehydrogenative coupling of benzaldehyde and benzimidazole without the use of transition metals (Scheme 31) [37,38]. The annulative coupling likely consists of four tandem reactions (Scheme 32): (a) an olefination, in which benzimidazole acts as a directing group; (b) an acetylation, in which benzimidazole acts as a leaving group and is replaced by acetate; (c) acetate hydrolysis with liberation of the COOH group; and (d) cyclization. 

**Scheme 33:**
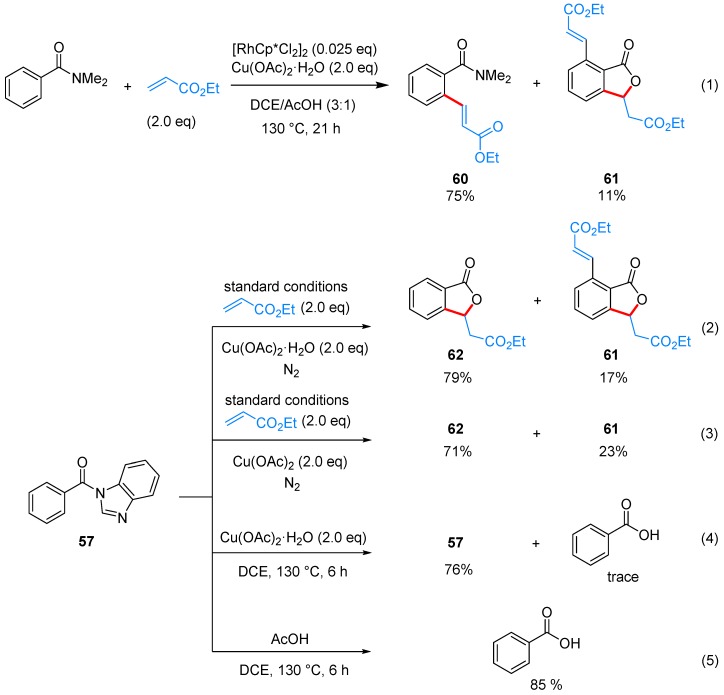
Control experiments for the Rh-catalyzed annulative coupling of *N*-benzoyl benzimidazole and electrophilic alkenes.

The double role of benzimidazole group has been elucidated by some control experiments. Treatment of *N*,*N*-dimethylbenzamide with ethyl acrylate affords olefinated compound **60** as the major product along with phthalide **61** (Scheme 33, (1)). This result suggests that benzimidazole acts as a directing group in the olefination reaction and as a leaving group in the following cyclization. The reaction of **57** and ethyl acrylate under nitrogen in the presence of Cu(OAc)_2_·H_2_O or Cu(OAc)_2_ provide similar results (Scheme 33, (2) and (3)), showing that neither air nor water are the source of oxygen needed to build the lactone ring. **57** is converted into benzoic acid when heated in the presence of Cu(OAc)_2_·H_2_O or acetic acid (Scheme 33, (4) and (5)), indicating that acetic acid is the source of oxygen.

By Ketone Hydroacylation [39,40,41]

**Scheme 34:**
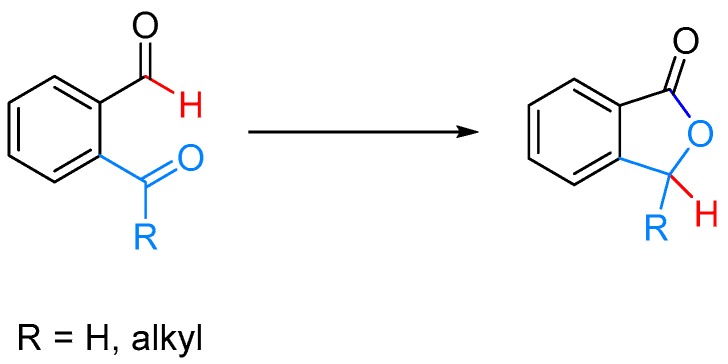
Intramolecular hydrogen transfer reaction in *o*-dialdehydes and *o*-ketobenzaldehydes to afford phthalide and 3-substituted phthalides, respectively.

An alternative approach to the construction of the γ-lactone ring of phthalides is the cyclization of *o*-dialdehydes or *o*-keto aldehydes by a hydroacylation reaction, in which the C—H bond of the aldehyde is added intramolecularly to the adjacent carbonyl group (Scheme 34). This method, involving only an atomic rearrangement, is redox-neutral and atom-economical. However, *o*-keto aldehydes, unlike *o*-phthalaldehyde, are not commercially available and need to be prepared, making this method multi-step.

Via C=O Hydrometalation (Tishchenko-Type Mechanism) [39]

**Scheme 35:**
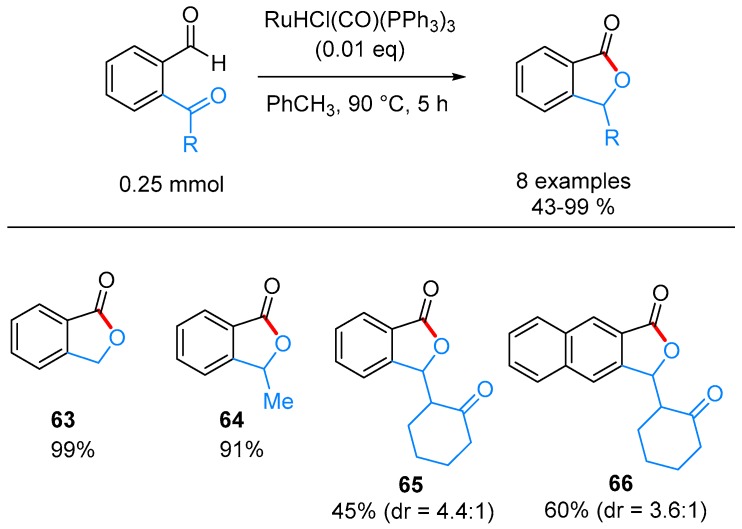
Ru-catalyzed hydroacylation of *o*-ketobenzaldehydes for the synthesis of 3-substituted phthalides.

**Scheme 36:**
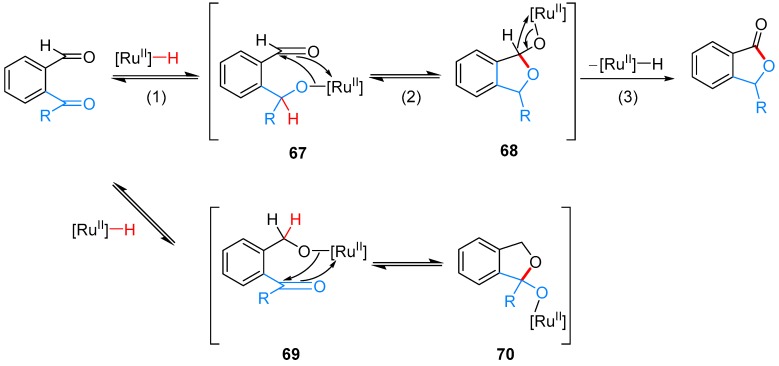
Proposed mechanism for the Ru-catalyzed hydroacylation of *o*-ketobenzaldehydes.

This method takes inspiration from the ability of group 8 metal hydrides, particularly ruthenium hydrides, to convert dialdehydes into lactones [42,43,44]. Phthalaldehyde is smoothly converted into phthalide upon heating in toluene in the presence of RuHCl(CO)(PPh_3_)_3_ as catalyst (Scheme 35, **63**). *o*-Acetyl benzaldehyde provides 3-methylphthalide **64**. Aromatic dialdehydes other than phthalaldehyde, aliphatic dialdehydes and ketoaldehydes also cyclize under such conditions, affording lactones other than phthalides. The reaction involves an intramolecular Tishchenko-type lactonization, which consists of three steps (Scheme 36): (1) hydroruthenation of the keto carbonyl group with formation of an alkoxyruthenium complex (**67**), (2) intramolecular alkoxy-ruthenation of the aldehyde carbonyl to provide acetal-type complex **68**, and (3) β-hydride elimination to liberate phthalide. Hydroruthenation also occurs at the aldehyde group giving alkoxyruthenium complex **69**, but this reaction is not productive because acetal **70** cannot undergo β-hydride elimination. Therefore, **69** goes back to **68**, which reacts irreversibly to form the phthalide. 

**Scheme 37:**
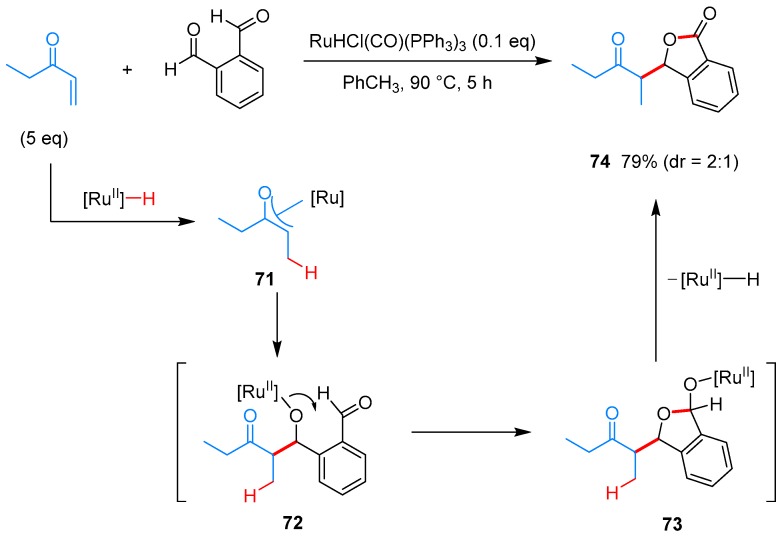
Cascade reaction of *o*-phthalaldehyde and an α,β-unsatuated ketone leading to a 3-substituted phthalide.

Substituents larger than methyl can be introduced in position 3 with no need for aldehyde pre-functionalization. When phthalaldehyde is reacted with an excess vinyl ketone (either linear or cyclic), the C=C double bond is reduced and the ketone is incorporated into the phthalide with formation of a bond between the lactone C-3 and the ketone C_α_, with modest diastereoselectivity (Scheme 35, compounds **65** and **66**, and Scheme 37, compound **74**). A large excess (5 eq) of ketone is necessary to suppress the competing intramolecular lactonization. For intermolecular reactions such as that in Scheme 37, hydroruthenation is a 1,4-addition to the α,β-unsaturated system rather than a 1,2-addition to carbonyl. Michael-type addition of Rh—H bond to vinyl ketone generates ruthenium enolate **71**. Nucleophilic attack of **71** to one formyl group of phthalaldehyde gives alkoxyruthenium complex **72**, which is analogous to **67**. Then, **72** undergoes nucleophilic addition to the adjacent formyl group to afford **73**. Finally, β-hydride elimination of **73** produces keto lactone **74**. The main advantage of this methodology is the possibility to introduce branched substituents in position 3 using commercially available starting materials (phthalaldehyde and α,β-unsaturated ketones), though not enantioselectively.

Via OC—H Oxidative Addition (Non-Tishchenko-Type Mechanism) [40]

**Scheme 38:**
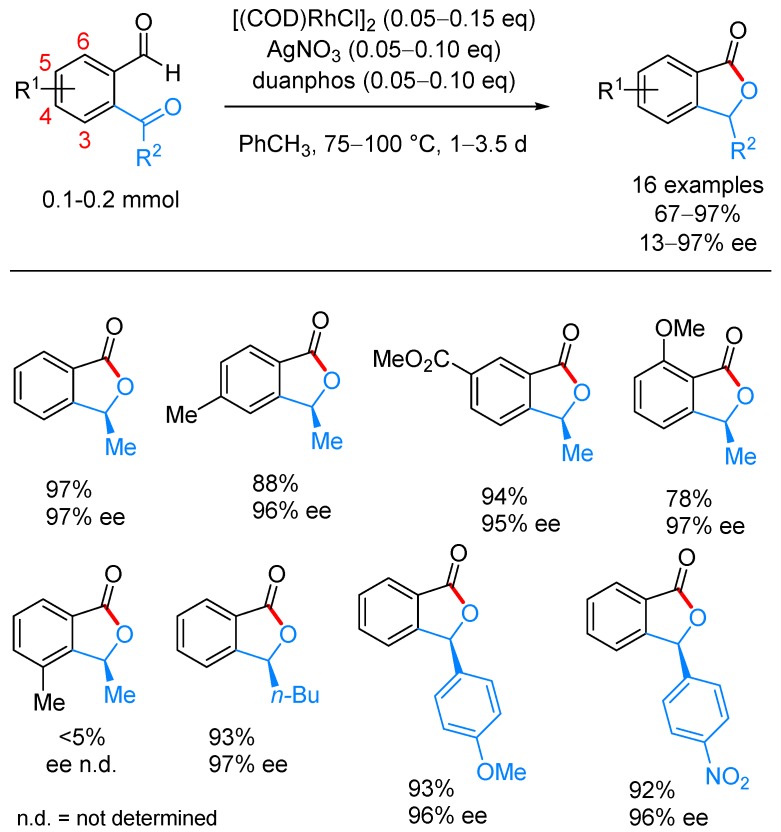
Rh-catalyzed hydroacylation of *o*-ketobenzaldehydes for the synthesis of 3-substituted phthalides.

An enantioselective version of ketone hydroacylation, with ees up to 97%, relies on cationic diphosphine Rh(I) complexes. 2-Acylbenzaldehydes cyclize to 3-substituted phthalides when heated in toluene in the presence of [Rh(COD)Cl]_2_, duanphos and AgNO_3_ (Scheme 38). Enantioselectivity is induced by the chirality of the active species, [Rh(duanphos)]^+^NO_3_^−^. Substituents in positions 4, 5, and 6 of the ring are tolerated, but steric hindrance in position 3 prevents reactivity. Ketones with substituents larger than methyl also react with high enantioselectivity but require higher catalyst loading.

**Scheme 39:**
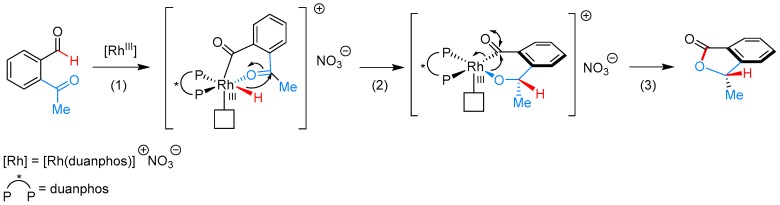
Mechanism of *o*-ketobenzaldehyde hydroacylation catalyzed by rhodium.

The catalytic cycle involves three steps (Scheme 39): (1) oxidative addition of Rh(I) to the aldehyde C—H bond, with formation of a Rh(III) hydride complex (C—H activation); (2) keto C=O insertion into Rh—H bond, to generate an alkoxyrhodium(III) complex; and (3) reductive elimination and lactonization to afford phthalide and regenerate the active species.

**Scheme 40:**
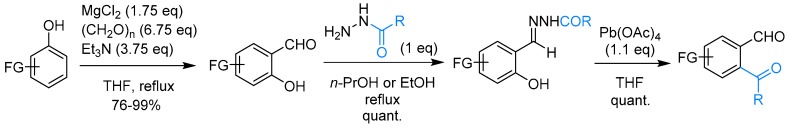
Multi-step synthesis of *o*-ketobenzaldehydes.

**Scheme 41:**
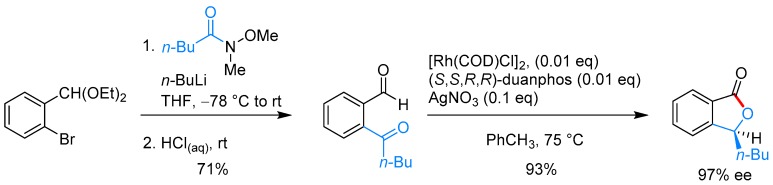
0.5-Gram-scale synthesis of celery extract (*S*)-3-butylphthalide.

The choice of counterion is critical for the reaction outcome and its effect is markedly substrate-specific. With alkyl aryl ketones, a moderately coordinating anion such as NO_3_^−^ must be used to maximize both yield and enantioselectivity. By occupying the vacant coordination site, nitrate ion plays a double effect: (1) it prevents CO deinsertion, suppressing a decarbonylative side reaction; and (2) it provides the proper arrangement of phosphine ligand necessary for enantioinduction. By contrast, biaryl ketones require non-coordinating anions such as mesylate or triflate, suggesting that one site must be left available for the π-coordination of phenyl ring or the approach of the sterically hindered substrate, or both. With respect to Ru(II) catalysis, the ketone hydroacylation catalyzed by Rh(I) offers the advantage of enantioselectivity; however, it requires additional steps to prepare the starting material (Scheme 40) [40], and one of such steps uses stoichiometric amounts of toxic Pb(OAc)_4_. This method has been applied to the 0.5 gram-scale synthesis of (*S*)-3-butylphthalide (Scheme 41).

Cobalt Catalysis [41]

**Scheme 42:**
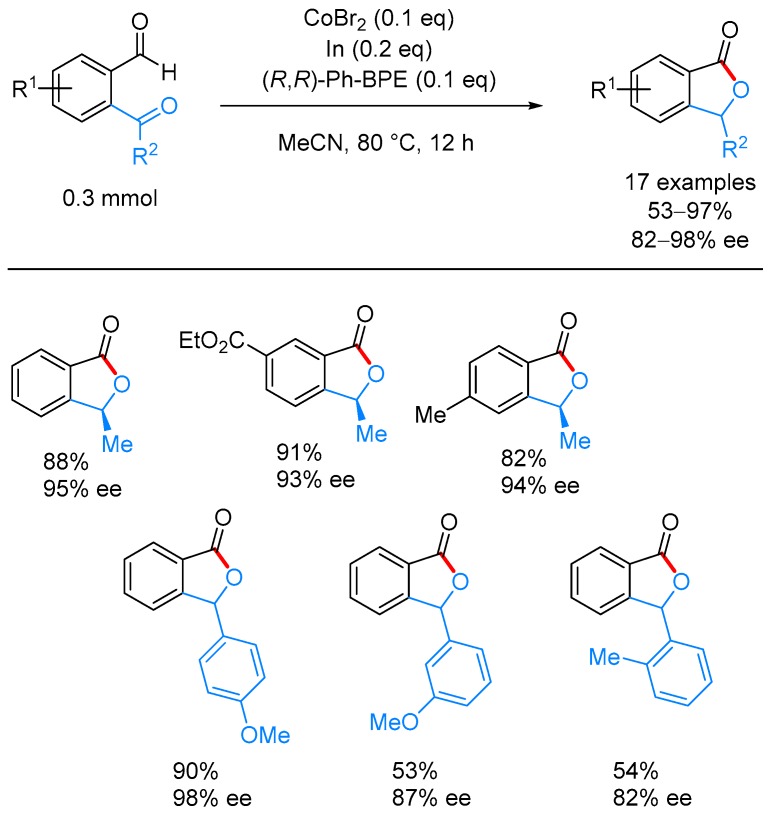
Co-catalyzed hydroacylation of *o*-ketobenzaldehydes to afford 3-substituted phthalides.

**Scheme 43:**
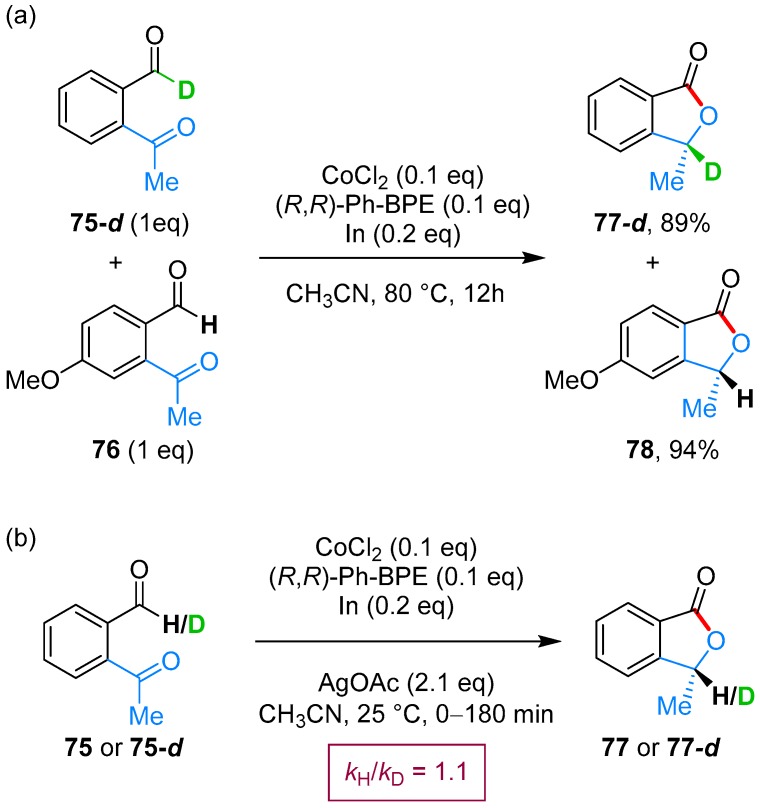
Deuterium-labelling experiments for the Co-catalyzed hydroacylation of *o*-ketobenzaldehydes.

**Scheme 44:**
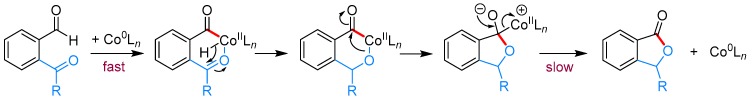
Outline of proposed mechanism for the Co-catalyzed hydroacylation of *o*-ketobenzaldehydes in homogeneous phase.

Another enantioselective approach uses a chiral diphosphine complex of Co(0), generated in situ by reduction of CoBr_2_ in the presence of indium metal (Scheme 42). (*R*,*R*)-Ph-BPE [(1,2-*bis*-(*R*,*R*)-2,5-diphenylphospholano)ethane] provides the highest enantioselectivity in this case. This method has a larger scope than that described above, because it can also be used to prepare indanones starting from 2-alkenylbenzaldehydes. The mechanism is also peculiar as it seems to be both heterogeneous and homogeneous. The mechanism in homogeneous phase has been elucidated by deuterium-labelling experiments. Under catalytic conditions, a mixture of deuterated ketobenzaldehyde **75-*d*** and non-deuterated ketobenzaldehyde **76** affords a mixture of deuterated phthalide **77-*d*** and non-deuterated phthalide **78** without any H/D crossover (Scheme 43a). This result shows that hydroacylation does not involve a free cobalt hydride species, and is therefore an intramolecular process that follows a non-Tishchenko-type mechanism. The reaction of 2-acetylbenzaldehyde shows no kinetic isotope effect (KIE ≈ 1, Scheme 43b), indicating that C—H activation is fast and not rate-determining. The reductive elimination is probably the slow step, because this step—unlike ketone insertion—does not involve the cleavage of a C—H bond. Based on deuterium-labelling experiments, a four-step mechanism can be proposed (Scheme 44): (1) fast oxidative addition of Co(0) to the aldehyde C—H bond; (2) hydrometalation of the keto carbonyl group; (3) intramolecular nucleophilic addition of alkoxide to the acyl group; and (4) slow (rate-determining) reductive elimination of Co(0) with generation of phthalide. Alongside the homogeneous mechanism, the reaction appears to follow a heterogeneous mechanism because it displays highly fluctuating yields (<5% at 12 h and 92% at 2 h for the reaction of 2-propenylbenzaldehyde-*d*), even when using the same batch of substrate and catalyst; this fluctuation implies non-reproducible kinetics that are indicative of the heterogeneous nature of catalyst.

### 2.2. 3,3-Disubstituted Phthalides

#### 2.2.1. 3-Ylidene Phthalides

From Benzoic Acids and Acrylates [45]

**Scheme 45:**
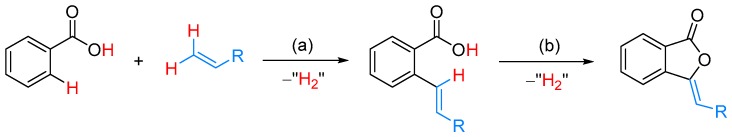
Cascade reaction of (**a**) Heck-type dehydrogenative alkenylation and (**b**) intramolecular dehydrogenative cyclization.

**Scheme 46:**
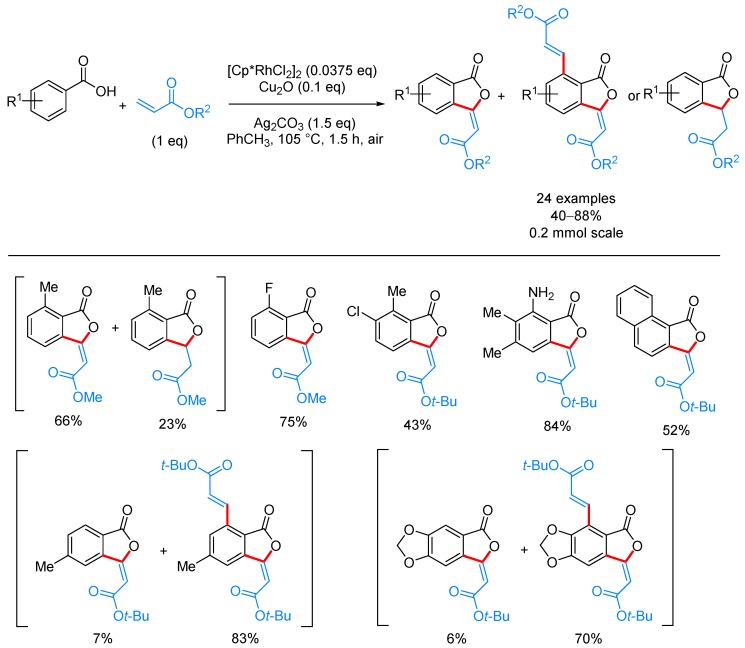
Rh-catalyzed annulative coupling of benzoic acids and acrylates for the synthesis of (*E*)-3-ylidenephthalides.

**Scheme 47:**
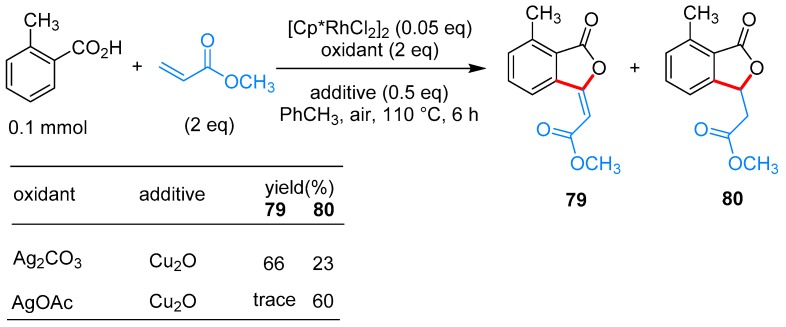
Oxidant and solvent effects in the Rh-catalyzed annulative coupling of benzoic acids and acrylates.

As seen above (pages 17–19), the annulative coupling of benzoic acids and acrylates produces 3-alkyl phthalides in the presence of a Rh(I) catalyst and a Cu(II) oxidant. When the same reaction is performed under a different set of conditions, i.e., using [Cp*RhCl_2_]_2_ as a catalyst, Ag_2_CO_3_ as an oxidant and Cu_2_O as an additive, the alkenylated intermediate undergoes an additional dehydrogenation and 3-ylidenephthalides are obtained as the major products, alongside minor amounts of 3-alkyl phthalides (Scheme 45 and Scheme 46). Analogously to the Rh^I^/Cu^II^ system, chemoselectivity depends on multiple factors such as reactants, oxidant anion and solvent. The yield of ylidenephthalide can be maximized, and that of alkyl phthalide minimized, by using sterically hindered *t*-butyl acrylate as the starting material. Conversely, 3-alkyl phthalides are almost the only product if the reaction is performed using methyl acrylate and AgOAc instead of Ag_2_CO_3_ (Scheme 47). If both *ortho*-positions are available for C—H bond activation, coupling occurs twice and a mixture of (*E*)-3-ylidenephthalides and (7*E*)-vinyl-(3*E*)-ylidenephthalides is obtained. In this case, the formation of disubstituted product can be maximized using an excess acrylate. The reaction is fully regioselective: the *E* isomer of 3-ylidene phthalide is obtained exclusively, and 3-substituted benzoic acids cyclize exclusively at the *para* position with respect to substituent. Remarkably, the reaction tolerates an unprotected amino group on the phenyl ring.

**Scheme 48:**
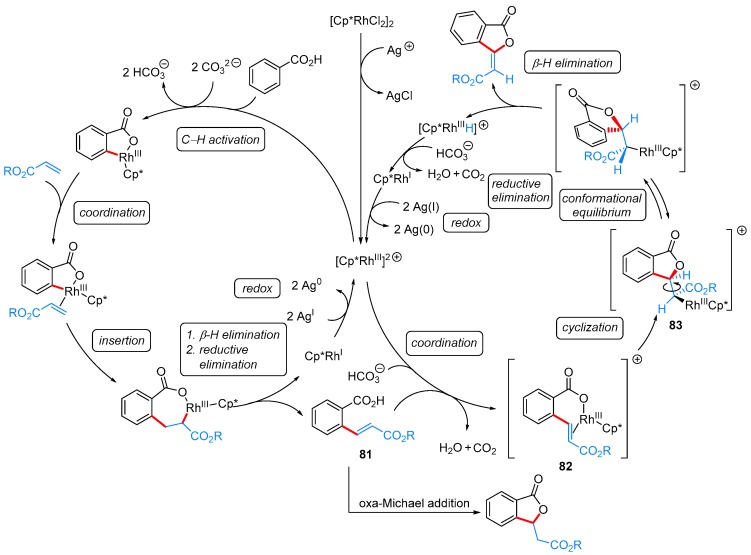
Proposed mechanism for the Rh-catalyzed annulative coupling of benzoic acids and acrylates leading to 3-ylidene phthalides.

The formation of 3-alkyl phthalide proceeds by a mechanism analogous to the one proposed for the Rh^I^/Cu^II^ system (Scheme 48); however, the *ortho*-alkenylated intermediate **81** can react by an alternative pathway leading to 3-ylidenephthalide. In this pathway, Rh(III) coordinates to the C=C double bond leaving the carboxylate group uncoordinated. Consequently, the Rh—O bond undergoes 1,2-addition to the C=C bond rather than 1,4-addition to the whole α,β-unsaturated system, and cyclization of **82** produces **83**. After conformational isomerization, **83** undergoes β-hydride elimination, releasing the *E*-isomer of the final product. Deprotonation of [Cp*Rh^III^H]^+^ generates Cp*Rh^I^, which is oxidized to [Cp*Rh^III^]^2+^ by Ag_2_CO_3_ to continue the catalytic cycle. According to this picture, the role played by Cu_2_O as an additive remains unclear. However, the effect of oxidant anion on chemoselectivity may be explained by the hard-soft acid-base theory [46,47,48]. If the oxidant is an effective chloride trapping agent (such as AgOAc), the active species is a highly unsaturated dicationic complex [(Cp*Rh^III^)^2+^], which behaves as a hard Lewis acid favoring oxygen coordination and hence 1,4-addition. In the absence of efficient chloride trapping agents such as Ag_2_CO_3_ (insoluble in toluene), the active species is a neutral and electron-rich complex (Cp*Rh^III^Cl_2_ monomer) that behaves as a soft Lewis acid favoring π-coordination and 1,2-addition. 

From Benzoic Acids and Vinyl Arenes [33,49]

**Scheme 49:**
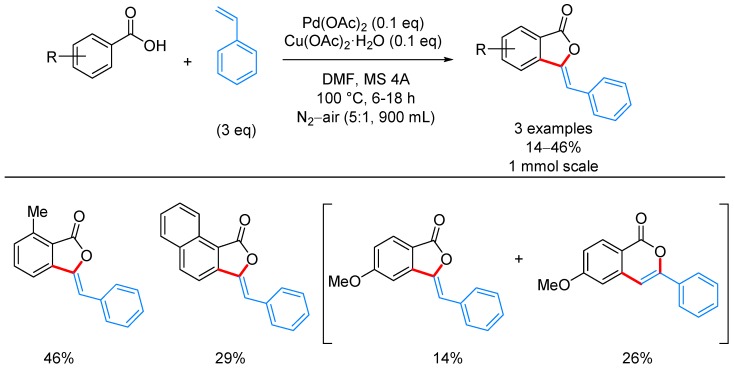
Pd-catalyzed annulative coupling of benzoic acids and vinyl arenes for the synthesis of (*Z*)-3-ylidenephthalides in the presence of Cu(II).

**Scheme 50:**
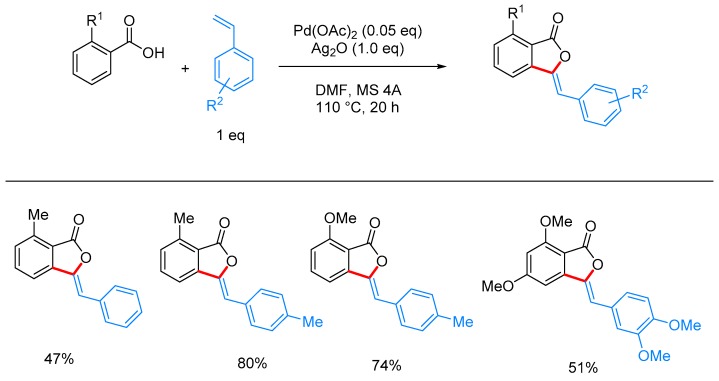
Pd-catalyzed annulative coupling of benzoic acids and vinyl arenes for the synthesis of (*Z*)-3-ylidenephthalides in the presence of Ag(I).

**Scheme 51:**
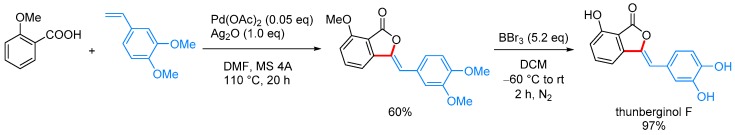
Total synthesis of thunberginol F.

Besides electron-poor alkenes, electron-neutral vinyl arenes can cyclize with benzoic acids to provide (*Z*)-3-ylidenephthalides. Two related methodologies have been developed, which use the same catalyst [Pd(OAc)_2_] and solvent (DMF) but a different oxidant [Cu(OAc)_2_·H_2_O [33] or Ag_2_O [49]] (Scheme 49 and Scheme 50, respectively). In both cases, the product structure depends on the substitution pattern of acid: *ortho*-substituted benzoic acids furnish 3-benzylidenephthalides only; unsubstituted benzoic acid affords 3-phenylisocoumarins only; *para*-substituted benzoic acids provide a mixture of phthalides and coumarins. Four hydrogens are removed during the coupling: two as water, two as dihydrogen. This methodology is strongly limited by the choice of benzoic acid, which needs an electron-donating substituent at the *para* position: benzoic acids with electron-withdrawing groups lead to unidentified products or undergo decarboxylative Heck-type coupling reactions. Such a reactivity pattern suggests an S_E_Ar mechanism for C—H bond activation. Furthermore, it is unclear whether the reaction works using vinyl arenes other than styrenes, because styrenes are the only alkenes used in this study. Despite the limited scope, this methodology is complementary to that reported above due to the opposite stereochemical outcome. The method has been applied to the synthesis of thunberginol on a 100-mg scale (Scheme 51).

**Scheme 52:**
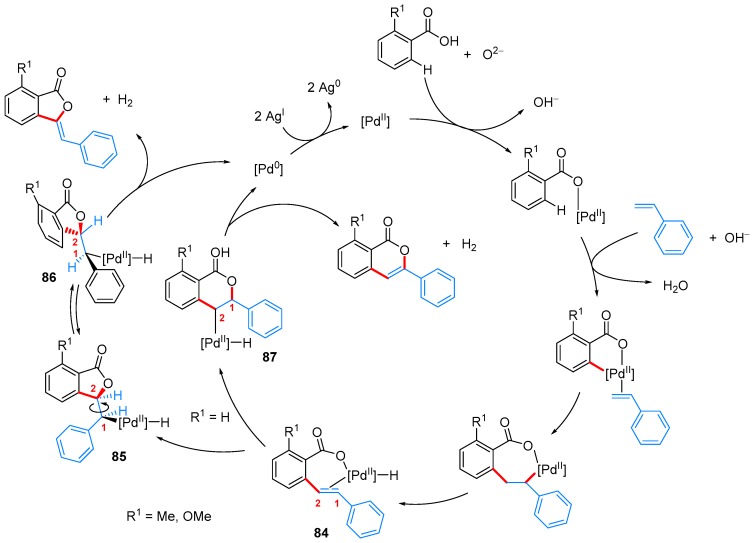
Proposed mechanism for the Pd-catalyzed annulative coupling of benzoic acids and vinyl arenes in the presence of Ag(I).

In the proposed catalytic cycle (Scheme 52), the *ortho*-alkenylated intermediate **84**—which is formed by the classical sequence of C—H bond activation, alkene coordination, insertion, and β-hydride elimination—can follow two pathways depending on the regiochemistry of addition of the O—Rh bond to the C=C double bond. For *ortho*-substituted benzoic acids, the attack of the coordinated O atom in **84** on C2 of the vinyl group generates the five-membered cycle **85**, which is in equilibrium with conformer **86**. **86** undergoes *syn* β-hydride elimination to afford (*Z*)-3-ylidenephthalide and PdH_2_, which is decomposed into H_2_ and Pd(0). For other benzoic acids, the attack of the O atom to C1 generates intermediate **87**, which then reacts similarly to **85** to give isocoumarin. The reasons for the preferential formation of **85** or **87** depending on ring substitution remain unknown. One possible explanation is a balance of kinetic and thermodynamic control. For *ortho*-substituted rings, the steric repulsion between the *ortho* substituent (or the *peri* hydrogen in the case of 1-naphtoic acid) and the carbonyl oxygen is smaller in phthalides than isocoumarins, thus favoring the formation of phthalides (kinetic control). In the case of unsubstituted ring, such a repulsion is absent and the six-membered cyclization, which is thermodynamically favored, occurs preferentially (thermodynamic control). The fact that *para*-substituted benzoic acids afford a mixture of products despite the absence of steric effects suggests that another factor is involved. 

From Benzoic Acids and Vinyl Acetates [50]

**Scheme 53:**
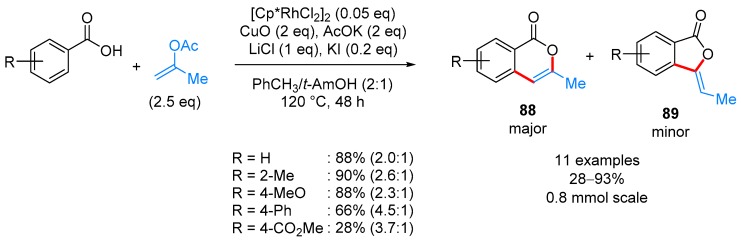
Rh-catalyzed annulative coupling of benzoic acids and isopropenyl acetate for the synthesis of 3-alkylisocoumarins and (*Z*)-3-alkylidenephthalides.

Electron-rich alkenes can also be coupled to benzoic acids to provide (*Z*)-3-ylidenephthalides, provided that geminal-substituted vinyl acetates are used (Scheme 53). Unfortunately, this methodology suffers from two limitations: (1) it provides phthalides as the minor products, the major products being isocoumarins; and (2) it is restricted to the introduction of a propylidene group on the lactone ring; vinyl acetates bearing substituents larger than methyl, such as *n*-Bu, 2-phenylethyl, and CO_2_Me afford isocoumarins exclusively. Despite the low yield and narrow scope, this method is useful because it provides access to phthalides that are not accessible by other methods.

**Scheme 54:**
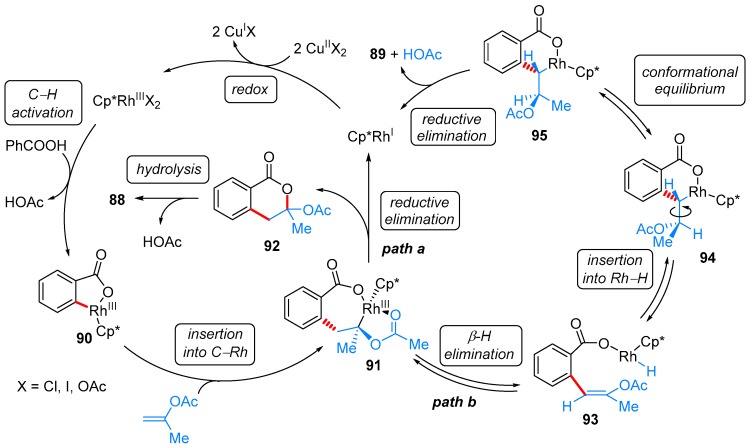
Proposed mechanism for the Rh-catalyzed annulative coupling of benzoic acids and vinyl acetates.

Isocoumarin **88** and phthalide **89** come from a common rhodabicycle intermediate (**91**), which is formed by insertion of the C=C double bond of isopropenyl acetate into the C—Rh bond of rhodacycle **90** (Scheme 54). **91** can follow two paths: in path a, reductive elimination generates **92**, which eliminates acetic acid to form isocoumarin **88**. In path b, β-hydride elimination affords vinylated intermediate **93**. Insertion of the C=C double bond of the vinyl group into the Rh—H bond gives 6-membered rhodacycle **94**, which is in equilibrium with its conformer **95**. *syn*-Elimination of acetic acid from **95**, followed by reductive elimination, affords phthalide **89**. Both paths a and b produce Cp*Rh^I^, which is oxidized by Cu^II^ to regenerate Cp*Rh^III^. The reversible nature of *ortho*-C—H bond activation is indicated by the partial deuteration of benzoic acid under catalytic conditions in the presence of deuterated *t*-AmOH.

**Scheme 55:**
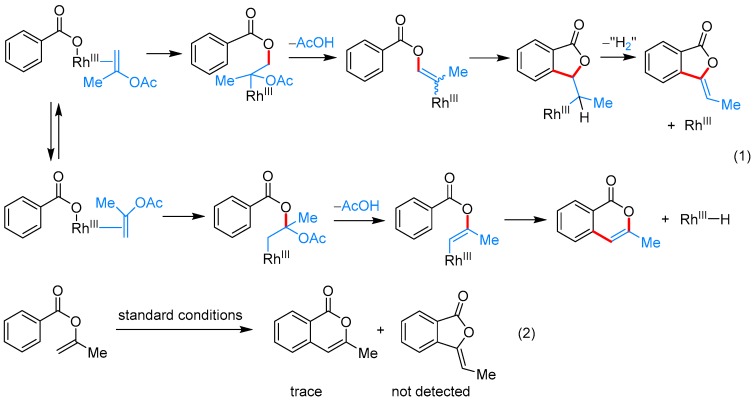
(**1**) Alternative mechanism for the Rh-catalyzed annulative coupling of benzoic acids and vinyl acetates via transvinylation. (**2**) Control experiment for the alternative mechanism.

An alternative mechanism involving transvinylation via alkene insertion to the Rh—O bond rather than Rh—H bond (Scheme 55, (1)) does not seem to operate because treatment of 2-propenyl benzoate under standard conditions affords only trace isocoumarin and no phthalide at all (Scheme 55, (2)).

From benzoic acids and alkynes [51]

**Scheme 56:**
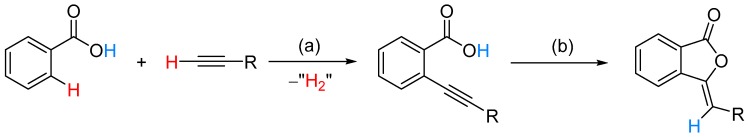
Cascade reaction of (**a**) Heck-type dehydrogenative alkynylation and (**b**) intramolecular cyclization.

Alkynes, being unsaturated, can serve as coupling partners of benzoic acids to make 3-ylidenephthalides, similarly to alkenes (Scheme 56). Because the reaction involves a dehydrogenation, terminal alkynes must be used. 

**Scheme 57:**
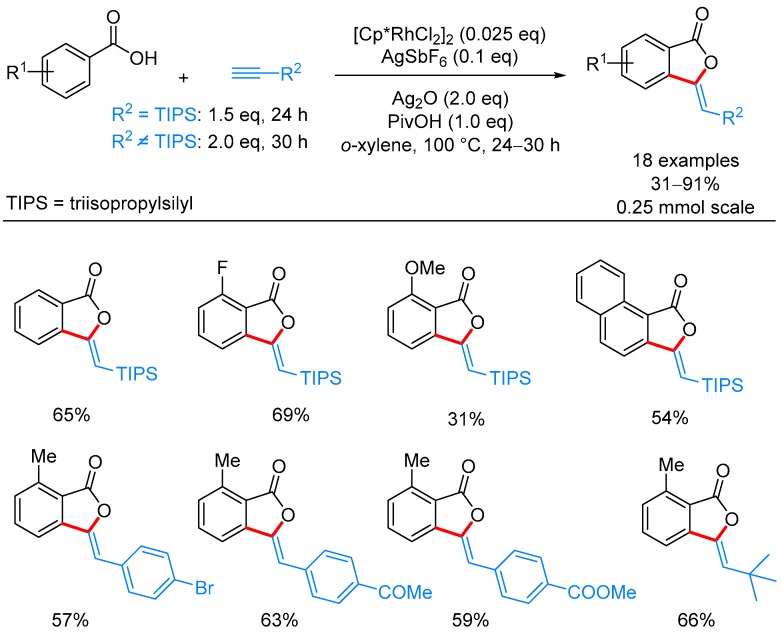
Rh-catalyzed annulative coupling of benzoic acids and terminal alkynes for the synthesis of (*Z*)-3-ylidenephthalides.

The reaction requires Ag_2_O as the oxidant and is catalyzed by [Cp*RhCl_2_]_2_ in *o*-xylene in the presence of pivalic acid (PivOH) as the additive (Scheme 57). This method, similarly to the coupling of vinyl arenes (pages 31–33), only affords the *Z* isomer of product, but has a much wider scope for two reasons: (1) it provides access to both 3-alkylidene and 3-arylidenephthalides, depending on whether an aliphatic or an aromatic alkyne is used; and (2) it works with *ortho*-unsubstituted benzoic acids (which yield isocoumarins when reacting with vinyl arenes under Pd(II) catalysis). Additionally, electron-rich and -poor substituents are tolerated on the phenyl ring of both acid and alkyne. Ester groups do not hydrolize, despite the release of 1 eq H_2_O during the reaction. 2-Methoxybenzoic acid is the only starting material among the tested substrates that affords the product in <50% yield. In each case, only trace isocoumarins are detected. 

**Scheme 58:**
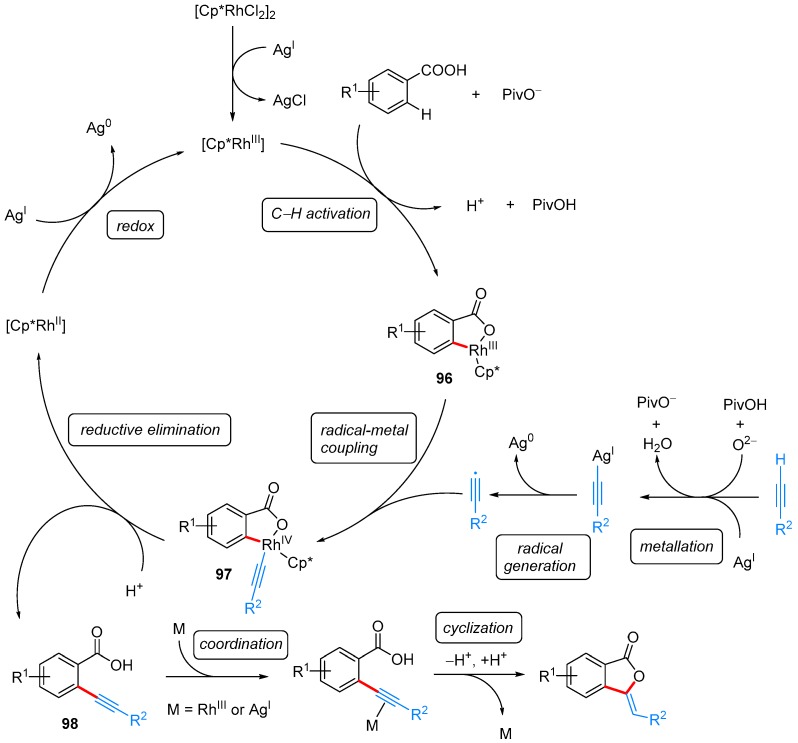
Proposed mechanism for the Rh-catalyzed annulative coupling of benzoic acids and alkynes.

The reaction likely follows a radicalic mechanism, as it is inhibited by radical scavengers such as TEMPO (2,2,6,6-tetramethylpiperidine-*N*-oxyl). According to the proposed mechanism (Scheme 58), alkyne coordination to Ag^+^ increases the terminal hydrogen acidity, thus favoring deprotonation by O_2_^−^ anion. The so formed silver(I) alkynyl splits homolytically into an alkyne radical (R—≡·) and Ag(0). The radical reacts with rhodacycle **96** to form the Rh(IV) complex **97**, which undergoes reductive elimination to give the alkynylated benzoic acid **98**. Cyclization of **98**, catalyzed by Rh(III) or Ag(I) or both, generates the final product. The released Rh(II) complex is oxidized by Ag(I) to regenerate the Rh(III) active species. The fact that no product is detected in the absence of additive or using PivOCs as an additive suggests that a proton source is needed to generate intermediate **98** and release the Rh(II) complex; the two *t*-Bu groups in PivOH make the acid soluble in a non-polar solvent such as *o*-xylene, while providing the steric hindrance necessary to prevent radical side reactions on the acid C_α_. Consistently with this hypothesis, 1,2-dichloroethane is not a good solvent for the reaction because it accepts hydrogen bonds from PivOH making it less available for the protonation of reaction intermediates.

From Benzoic Acid and Anhydrides [52]

**Scheme 59:**
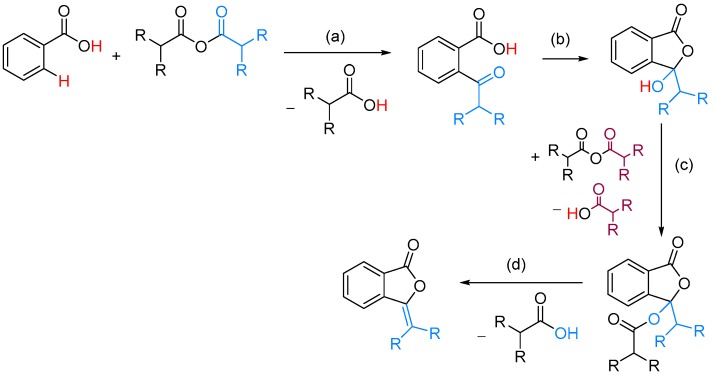
Cascade reaction of (**a**) dehydrogenative acylation, (**b**) acylalization, (**c**) acetylation, and (**d**) β-elimination to afford 3-ylidenephthalides.

Anhydrides can serve as precursors of acyl groups in the acylation of benzoic acids (Scheme 59a). The *o*-keto acid so obtained cyclizes into an acylal (b), which is acylated (c) and eventually eliminates AcOH to afford ylidene phthalide (d). Overall, two anhydride molecules are used up: one of the four acyl groups is incorporated into the lactone ring, the remaining three are eliminated as acid while achieving dehydrogenation. 

**Scheme 60:**
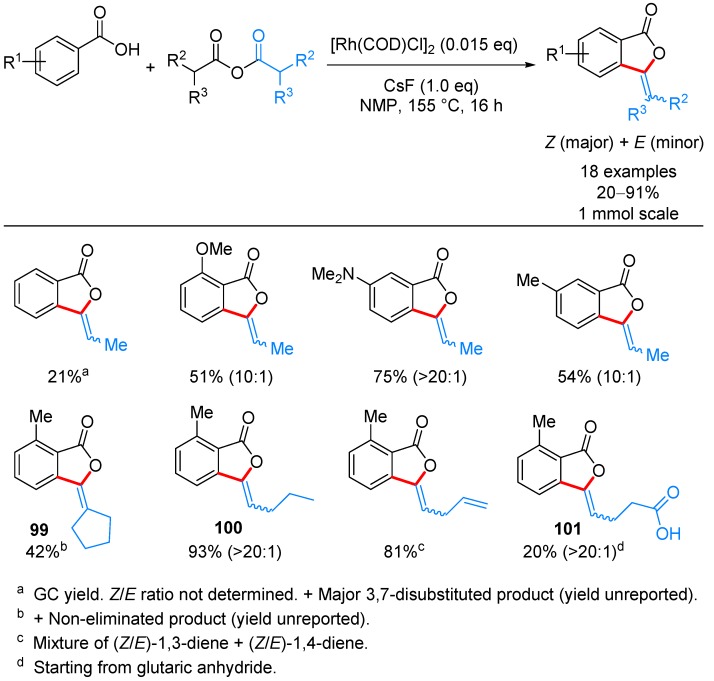
Rh-catalyzed annulative coupling of benzoic acids and anhydrides for the synthesis of 3-ylidenephthalides.

The optimal catalytic system is a combination of [(COD)RhCl]_2_ (COD = 1,5-cyclooctadiene) and CsF in NMP (Scheme 60). *Ortho*- and *meta*-substituted benzoic acids afford 3-ylidenephthalides as a mixture of regioisomeric products, with a marked selectivity for the *Z* isomer (*Z*/*E* ≥ 10:1); *para*-substituted and unsubstituted benzoic acids mainly provide the double-substitution product (7-substituted 3-ylidenephthalide). For *meta*-substituted benzoic acids, cyclization takes place selectively at the less hindered *ortho* position. The reaction has only been investigated with aliphatic anhydrides derived from propionic acid or longer-chain acids; neither acetic anhydride nor aromatic anhydrides have been tested.

**Scheme 61:**
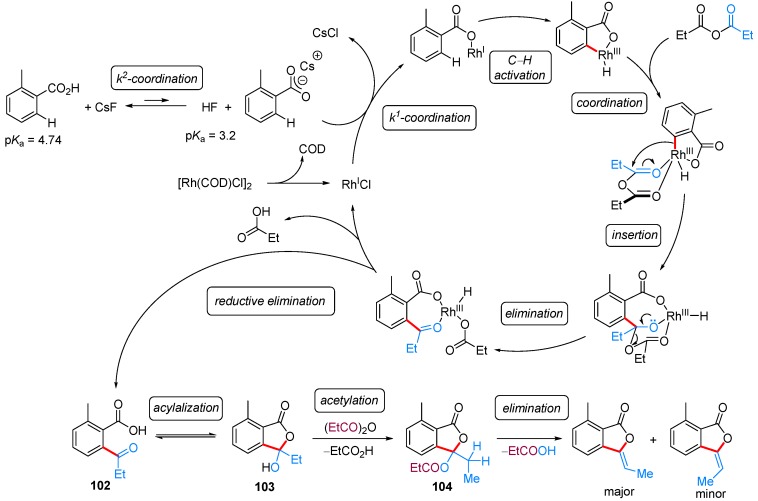
Possible mechanism for the Rh-catalyzed annulative coupling of benzoic acids and anhydrides.

**Scheme 62:**
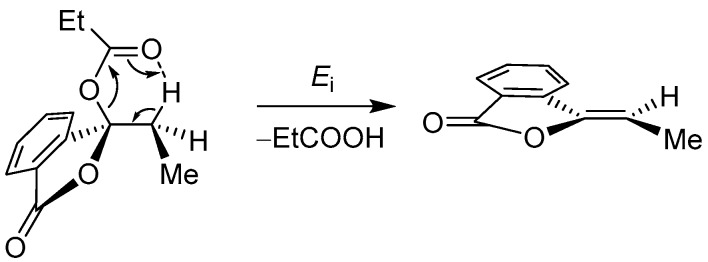
Possible mechanism for the elimination step of **104**.

The reaction mechanism via *ortho*-acylation (Scheme 61) is supported by three pieces of evidence: (1) the precursor of the final product, **104**, can be isolated if the reaction is performed under a set of conditions that do not favor elimination, for instance using branched aliphatic aldehydes in non-polar solvents (mesytilene) and a weak base (aqueous NaHCO_3_) for work-up; (2) the *ortho*-acylbenzoic acid **102** is isolated as the major product if the reaction is performed in a non-polar solvent (mesytilene) and worked up with a strong base (hot aqueous NaOH), which hydrolizes the acetate **104** [53]; and (3) this mechanism explains the reactivity trend observed for anhydrides: for branched aliphatic anhydrides, the final elimination step is kinetically unfavored due to the steric hindrance of the 3,3-disubstituted precursor, and the elimination product is obtained together with the non-eliminated product (**99**). Unsaturated anhydrides partially isomerize after coupling to form the conjugated double-bond isomer (**100**). Cyclic anhydrides such as glutaraldehyde open up releasing a carboxyl group at the opposite end of the ylidene chain (**101**). This method is not limited to the use of simple anhydrides: commercially unavailable anhydrides can be generated in situ from the reaction of an acid and a simple, commercially available anhydride such as propionic or pivaloyl anhydride. The observed *Z* selectivity is likely determined by the stereochemistry of the final elimination step, which mainly occurs intramolecularly with the methyl group and the phenyl ring in the *trans* position to minimize steric repulsion (Scheme 62).

#### 2.2.2. 3-Alkyl-3-Vinyl Phthalides

From Benzoic Acids and Allenes [54]

**Scheme 63:**
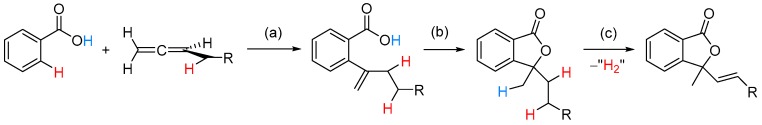
Cascade reaction of (**a**) redox-neutral alkenylation, (**b**) intramolecular cyclization, and (**c**) dehydrogenation to afford 3,3-disubstituted phthalides.

**Scheme 64:**
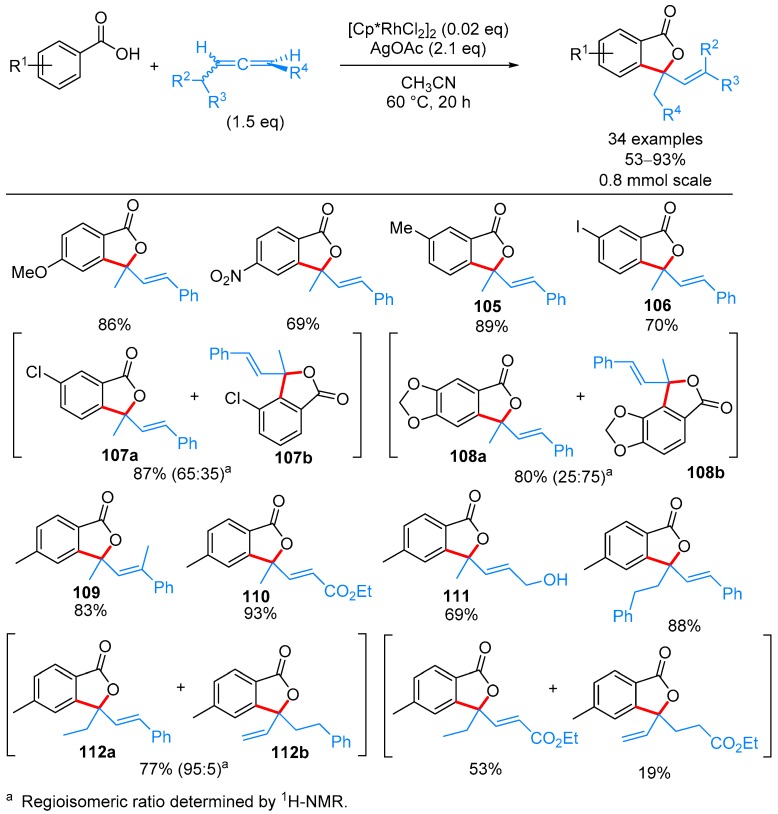
Rh-catalyzed annulative cyclization of benzoic acids and allenes for the synthesis of 3,3-disubstituted phthalides.

Terminal allenes react with benzoic acid in a [4 + 1] cycloaddition reaction involving three changes: (1) the *sp*-hybridized carbon atom is incorporated into the lactone ring; (2) the terminal C=C double bond is reduced; and (3) the internal C=C bond shifts to the adjacent position (Scheme 63 and Scheme 64). The product is a 3-alkyl-3-vinyl phthalide with the vinyl group in the *trans* configuration. 

The combination of CH_3_CN and AgOAc is critical for the reaction outcome; temperatures >50 °C are also needed for efficient C—H activation. Substituents on the phenyl ring do not affect reactivity; however, they do control regiochemistry when located in position 3: bulky substituents such as methyl and iodine, or the strongly electronegative fluorine atom, prevent C—H bond activation on the adjacent carbon, leading to only one regioisomer (**105** and **106**). Small-size, electron-donating groups by mesomeric effect deliver two regioisomeric products (**107a + 107b** and **108a + 108b**). The allene can be branched (**109**) or functionalized (**110** and **111**). Internal allenes can also be used. If substituents on both sides of the allene system are different, the reaction affords two regioisomeric products, because the C=C double bond can migrate to two different positions. When two products with largely different stabilities are possible, the reaction is regioselective and the thermodynamically more stable product predominates, as the reaction is under thermodynamic control. For instance, product **112a** with an internal, conjugated C=C double bond predominates over **112b** having an external, unconjugated C=C bond. The effect of allene stereochemistry on the product chirality has not been investigated because allenes have been used as racemates. 

**Scheme 65:**
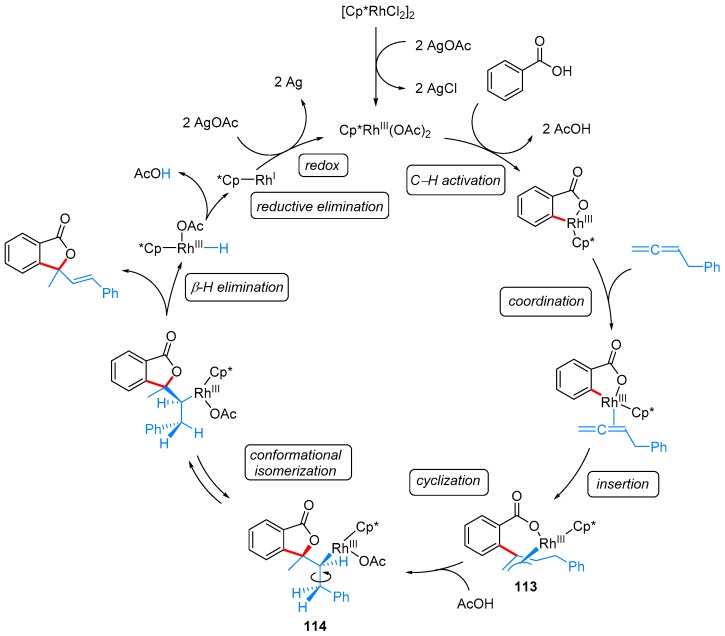
Proposed mechanism for the Rh-catalyzed annulative coupling of benzoic acids and allenes.

The catalytic cycle (Scheme 65) features the formation of a π-allylic rhodacycle **113**, obtained by insertion of an allene double bond into the C—Rh bond. Intramolecular nucleophilic addition of the coordinated oxygen atom to the π-allyl system of **113** followed by protonation gives intermediate **114**. After conformational isomerization, β-hydride elimination of **114** affords the final product with a C=C double bond in the *trans* configuration, alongside a Rh(III)-hydride complex. Eventually, reductive elimination, followed by reoxidation of Rh(I) to Rh(III) by AgOAc, regenerates the active species.

**Scheme 66:**
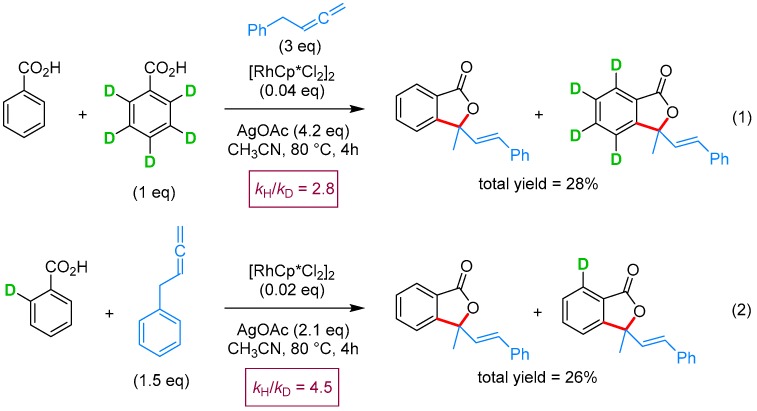
Deuterium-labelling experiments for the Rh-catalyzed annulative coupling of benzoic acids and electrophilic alkenes.

The large values (>2.5) of kinetic isotope effect measured for both intermolecular and intramolecular competition reactions (Scheme 66, (1) and (2)) suggest that C—H bond activation is the rate-limiting step.

#### 2.2.3. 3,3-Dialkyl Phthalides

From Benzoic Acids and Internal Alkynes [55] 

**Scheme 67:**
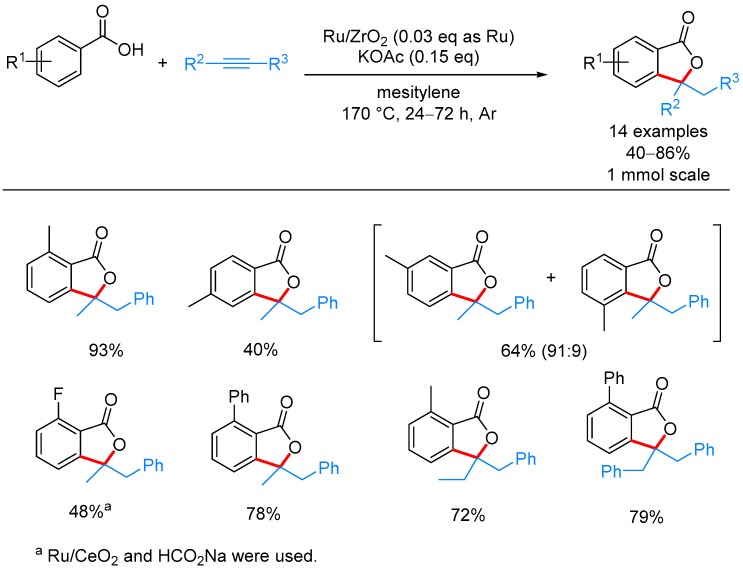
Ru-catalyzed annulative coupling of benzoic acids and internal alkynes in heterogeneous phase for the synthesis of 3,3-disubstituted phthalides.

Internal alkynes of type R—C≡C—Ph (R = alkyl or Ph) can be coupled to benzoic acid to produce 3,3-disubstitued phthalides in the presence of a ZrO_2_-supported ruthenium catalyst (ruthenium loading = 2.0 wt%) (Scheme 67). The reaction does not require any oxidant because it is not a dehydrogenation: the two hydrogen atoms removed from benzoic acid are used for alkyne reduction. The reaction of 1-phenylpropyne only affords the regioisomer with the phenyl ring located far from the spiranic center. 

**Scheme 68:**
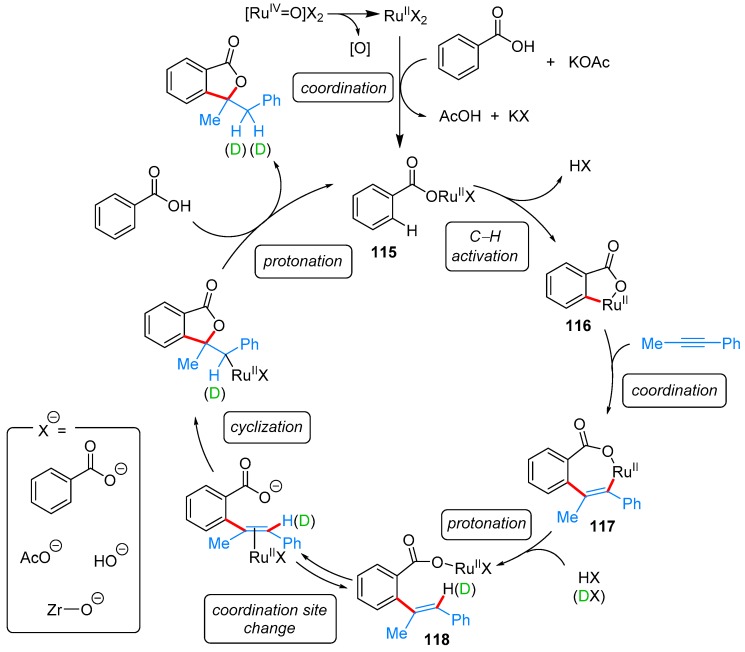
Proposed mechanism for the Ru-catalyzed annulative coupling of benzoic acids and internal alkynes in heterogeneous phase.

The surface of ZrO_2_ is coated with a Ru(IV)-oxo species, which is reduced to Ru(II) under reflux (Scheme 68). The Ru(II) active species undergoes ligand exchange with benzoic acid to form ruthenium benzoate **115**; this step is accelerated by the addition of KOAc. After *ortho*-ruthenation, alkyne inserts into the Ru—C bond of **116** regioselectively forming **117**, then is protonated to produce **118**. A change in rhodium coordination site from O to C=C double bond triggers an intramolecular cyclization via 1,2-addition; eventually, protonation produces phthalide and regenerates ruthenium benzoate **115**.

**Scheme 69:**
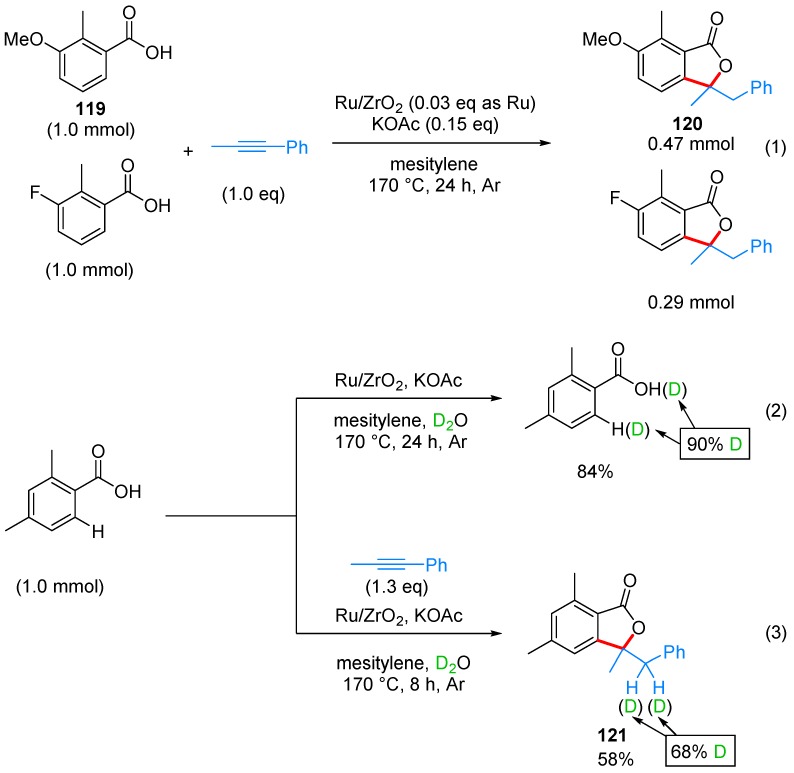
Deuterium-labelling experiments for the Ru-catalyzed coupling of benzoic acids and internal alkynes in heterogeneous phase.

Traces of alkenylated intermediate **118** can be trapped by methylation with MeI/K_2_CO_3_ in DMF. In an intermolecular competition experiment (Scheme 69, (1)), benzoic acid **119** bearing an electron-donating substituent is preferentially converted to the corresponding phthalide **120**, suggesting that C—H bond activation is the rate-determining step and proceeds by an electrophilic aromatic substitution mechanism.

Further mechanistic information is provided by deuterium-labelling experiments (Scheme 69, (2) and (3)). If 2,6-dimethylbenzoic acid is treated with D_2_O under catalytic conditions, a significant amount of deuterium is incorporated at the *ortho*-position, suggesting that ruthenation is a reversible process (Scheme 69, (2)). If the same reaction is performed in the presence of 1-phenylpropyne, the methylene group of product **121** is also deuterated, indicating that protonation of ruthenacycle **117** is also reversible (Scheme 69, (3)).

Two pieces of evidence suggest that a homogeneous mechanism operates alongside the heterogeneous mechanism: (1) if the supported ruthenium catalyst is removed from the reaction mixture, further reaction progress is retarded but not stopped; and (2) the reaction also works using [RuCl_2_(*p*-cymene)]_2_ as a homogeneous catalyst in the place of Ru/ZrO_2_ with comparable yield (82 *vs* 93% for the reaction of *o*-toluic acid and 1-phenylpropyne).

### 2.3. Unsubstituted Phthalide

#### 2.3.1. By Annulative Disproportionation of *o*-Phthalaldehyde

Tishchenko-Type Reactions [56,57,58]

**Scheme 70:**
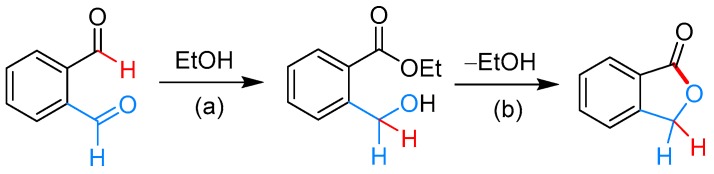
Cascade reaction of (**a**) aldehyde disproportionation and (**b**) intramolecular cyclization for the synthesis of unsubstituted phthalide.

**Scheme 71:**
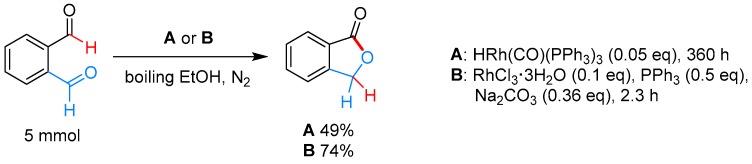
Rh-catalyzed hydroacylation of *o*-phthalaldehyde in ethanol for the synthesis of unsubstituted phthalide.

Esters, including lactones such as phthalide, can be prepared from aldehydes by a Tishchenko-type disproportionation reaction. In such a reaction, a hydrogen atom is transferred intramolecularly from an aldehyde (hydrogen donor) to another (hydrogen acceptor; Scheme 70). In addition to RuHCl(CO)(PPh_3_)_3_ (Scheme 35, page 24), both [RhH(CO)PPh_3_] and a catalyst made in situ from RhCl_3_·3H_2_O, PPh_3_ and Na_2_CO_3_ catalyze the reaction (Scheme 71) [56]. Alcohol also participates in the hydrogen transfer process. 

**Scheme 72:**
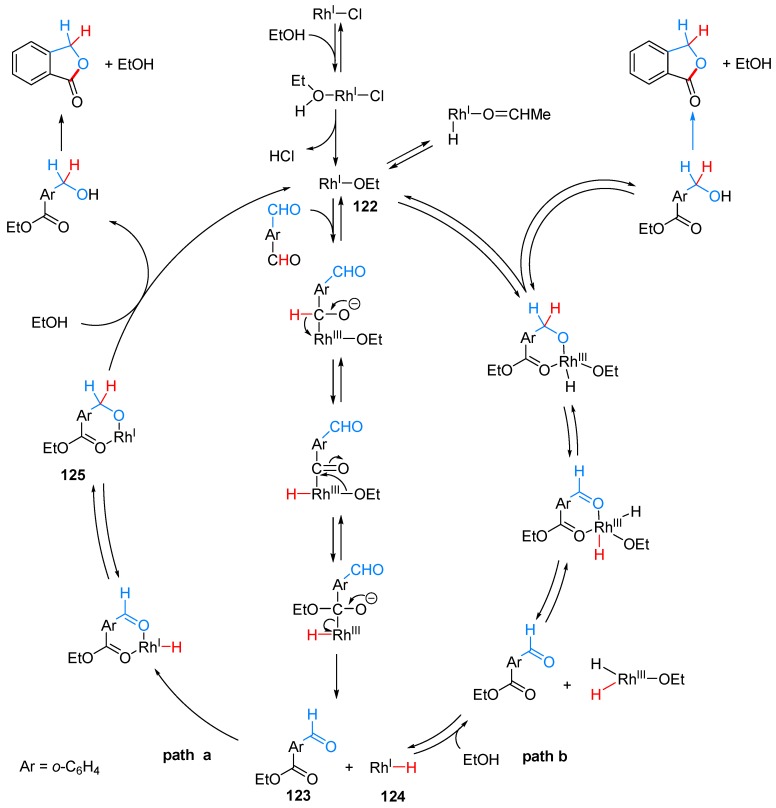
Proposed mechanism for the Rh-catalyzed hydroacylation of *o*-phthalaldehyde in ethanol.

The active species is a Rh(I) ethoxide complex (**122**) generated by reaction of Rh(I)Cl and EtOH (Scheme 72). **122** acts as a hydride sink towards aldehyde, in a sequence of steps whose overall effect is an EtO^−^/H^−^ exchange resulting into the oxidation of phthalaldehyde into ester **123**. Then, two pathways are available for the formation of product. In path a, Rh(I)H **124** transfers the hydride ion to another aldehyde forming an alkoxide (**125**), which is protonated and released by the excess alcohol present as solvent, while the Rh(I)OEt complex is regenerated. 

In path b, Rh(I)H undergoes oxidative addition to EtOH; the Rh(III) complex so formed coordinates to the formyl ester, then transfers a hydride ion to the aldehyde group; eventually, the alcohol is reductively eliminated. The ester and alcohol formed from path a or b cyclize to afford the lactone and regenerate ethanol. Although in principle both oxidation and reduction can take place twice in the same molecule, no diester or dialcohol are observed. This result suggests that the Rh(I)H complex is transferred intramolecularly from one carbonyl group to another, rather than being released in the solvent.

**Scheme 73:**
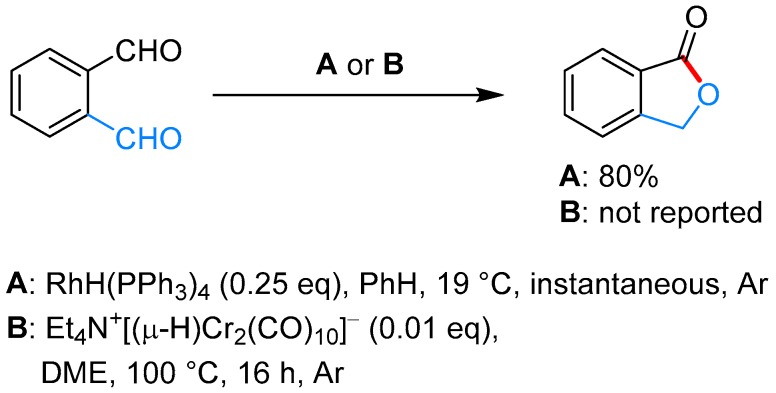
Rh- and Cr-catalyzed hydroacylation of *o*-phthaldehyde in aprotic solvents for the synthesis of unsubstituted phthalide.

Two analogous catalytic systems have been developed to achieve the conversion of *o*-phthalaldehyde into phthalide by a Tishchenko-type mechanism (Scheme 73): (**A**) RhH(PPh_3_)_4_, which is commercially available and provides an instantaneous reaction at 19 °C due to the catalyst geometry favorable for complexation of the aldehyde carbonyls [57]; and (**B**) the dimeric chromium(0) complex Et_4_N^+^[(μ-H)Cr_2_(CO)_10_]^−^ [58], obtainable by reduction of Cr(CO)_6_ with potassium/graphite (C_8_K) [59]. Both catalysts, containing a hydride ligand, do not require a protic solvent to effect hydrogen transfer.

Non-Tishchenko-Type Reactions [60] 

**Scheme 74:**
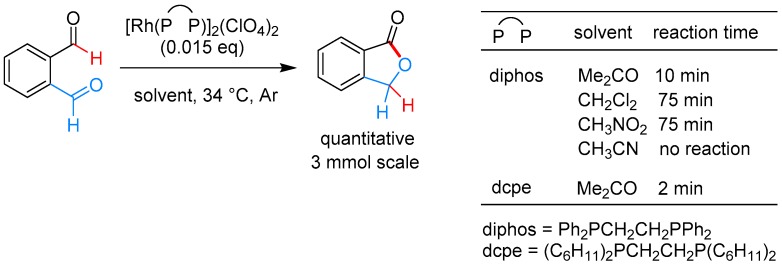
Rh-catalyzed hydroacylation of *o*-phthalaldehyde in acetone for the synthesis of unsubstituted phthalide.

Rhodium-phosphine complexes lacking of hydride ligands can also catalyze the disproportionation of *o*-phthalaldehyde by a non-Tishchenko-type reaction (Scheme 74) [60]. The most plausible mechanisms for this reaction are: (1) C—H oxidative addition, olefin-hydride insertion, and reductive elimination; or (2) C—H oxidative addition, carbonyl insertion into the metal-acyl bond, and reductive elimination. For either mechanism, at least three vacant metal sites are required, one each for the hydride, the acyl ligand, and the aldehyde. This requirement is satisfied using highly unsaturated Rh(I) complexes of the type [Rh(diphosphine)]^+^ and weakly coordinating anions (ClO_4_^−^). The choice of solvent is critical for catalytic activity, because solvent affects not only the coordination number of metal but also the aggregation state of complex (Scheme 74). In acetone, the diphos-Rh complex exists mainly as the disolvento monomer [Rh(diphos)(acetone)_2_]^+^, and the acetone ligands are easily displaced by substrate, leading to relatively high turnover frequency (TOF ~10^−1^ s^−1^). In CH_2_Cl_2_ and CH_3_NO_2_ the catalyst exists as the aryl-bridged dimer, which must be split by substrate before catalysis can occur. As a consequence, turnover frequency is lower in these solvents (TOF ~10^−2^ s^−1^). In CH_3_CN the catalyst exists as a disolvento monomer [Rh(diphos)(CH_3_CN)_2_]^+^ (as in the case of acetone) but the acetonitrile ligands are not displaced by carbonyl groups, so no catalysis occurs in this solvent. In methanol solution the catalyst exists as [Rh(diphos)(CH_3_OH)_2_]^+^, and the solvent ligands are easily replaced, but the formation of phthalide is slow because most of the aldehyde is tied up as hemiacetal and acetal. Stereoelectronic properties of diphosphine also affect catalysis: large chelate ring size and electron-donating groups are beneficial, with dcpe being the most effective ligand among those tested. Catalysis with [Rh(dcpe)]ClO_4_ in acetone is very rapid, occurring at even −60 °C and being complete within 10 min.

**Scheme 75:**
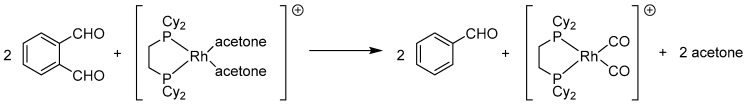
Decarbonylative side-reaction of *o*-phthalaldehyde in the presence of [Cy_2_PCH_2_CH_2_PCy_2_Rh(acetone)_2_]^+^. Cy = cyclohexyl.

Besides solvent and phosphine ligand, catalyst concentration and substrate:catalyst ratio need to be controlled. Rhodium concentration must be kept as low as ~10^−3^ M in order to avoid a decarbonylative side reaction, which leads to catalyst deactivation (Scheme 75). 

**Scheme 76:**
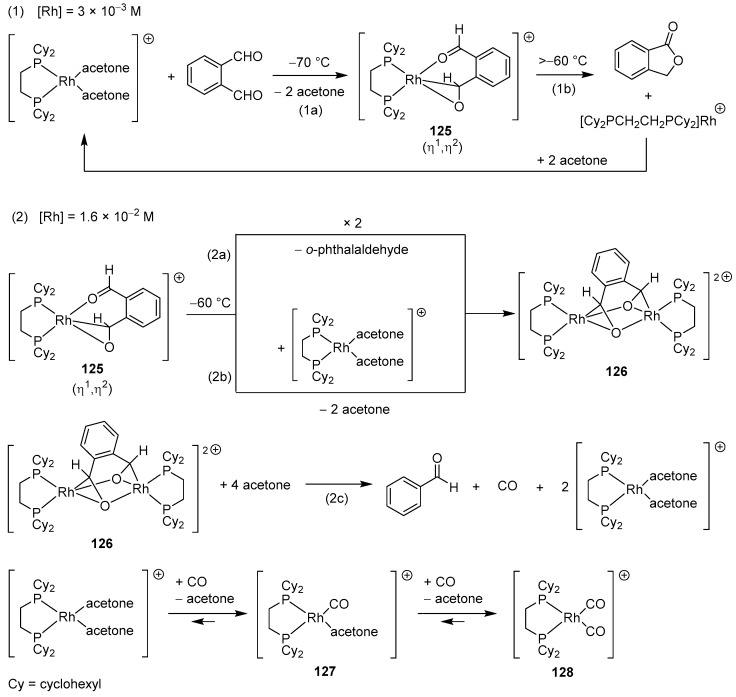
Reaction intermediates in (**1**) the hydroacylation of *o*-phthalaldehyde at low rhodium concentrations and (**2**) the decarbonylation of *o*-phthalaldehyde at high rhodium concentrations, followed by catalyst deactivation.

At −70 °C, a stable (η^1^,η^2^-dialdehyde)rhodium adduct (**125**) can be isolated from the stoichiometric reaction of *o*-phthaldehyde and [Rh(dcpe)(acetone)_2_]ClO_4_ (Scheme 76, (1a)). Above −60 °C, at low rhodium concentrations, the complex disappears and the lactone begins to form (Scheme 76, (1b)). Above −60 °C and at high rhodium concentrations, **125** dimerizes into **126** (Scheme 76, (2a) and (2b)), which catalyzes the decarbonylation of *o*-phthalaldehyde (Scheme 76, (2c)). CO displaces acetone almost irreversibly forming **127** and **128**, which are highly stable and therefore inactive. Decarbonylation is also observed at >200:1 substrate:Rh ratios, although lactonization is still the major reaction in this case. It remains unclear why the rate of decarbonylation increases with increasing rhodium concentration and substrate:Rh ratio. 

#### 2.3.2. By Oxidation of 1,3-dihydroisobenzofuran in Homogeneous Phase

**Scheme 77:**
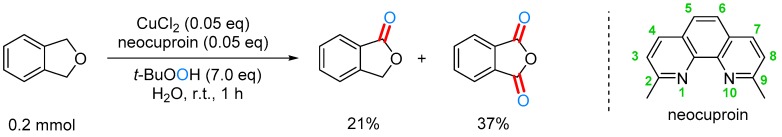
Cu-catalyzed oxidation of 1,3-dihydroisobenzofuran in homogeneous phase for the synthesis of phthalide and phthalic anhydride.

Unsubstituted phthalide can be prepared by treating phthalan (1,3-dihydroisobenzofuran) with *t*-BuOOH (*tert*-butylhydroperoxide) in water in the presence of neocuproin and a Cu(II) halide (Scheme 77) [61]. Various aryl alkanes, both oxygenated and non-oxygenated, can be oxidized using this method. Substrates with a single methylene group are converted to the corresponding carbonyl compounds selectively; substrates with more than one methylene group, including phthalan, can be oxidized twice. Thus, the oxidation of phthalan provides a mixture of phthalide and phthalic anhydride in which phthalide is the minor product (<30% yield). The low chemoselectivity and yield are the main drawbacks of this methodology. Among the advantages, the reaction involves selective allylic (sp^3^)C—H bond activation in the presence of (sp^2^)C—H bonds, which are usually more reactive. Additionally, this method can be considered eco-friendly for three reasons: (1) it uses a safe catalyst (Cu^II^), solvent (H_2_O), and oxidant (an organic peroxide); (2) it runs under mild conditions (room temperature); and (3) the product can be isolated by simple extraction with ethyl acetate, allowing the catalyst reuse. No leaching of the ligand neocuproine into the organic phase is observed.

Cu(I) and Cu(II) salts are equally effective in catalyzing the reaction, suggesting that Cu(I) is oxidized to Cu(II) in situ. In contrast, anion plays a significant effect on reactivity: copper halides (irrespective of halogen) perform much better than Cu(NO_3_)_2_ and CuSO_4_, indicating that moderately coordinating ligands are needed for catalytic activity [62]. The methyl groups in positions 1 and 9 on neocuproin are also crucial: if they are removed or placed in other positions on phenanthroline ring, both reactivity and selectivity drop drastically.

**Scheme 78:**
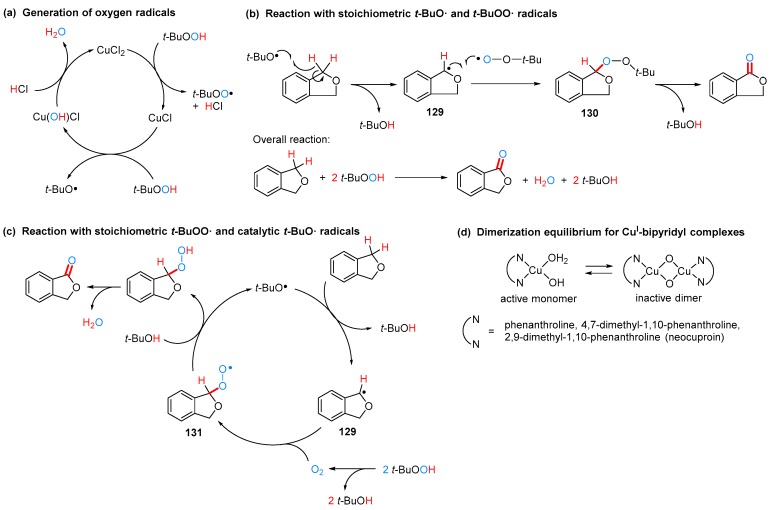
Proposed mechanism for Cu-catalyzed oxidation of 1,3-dihydroisobenzofuran in homogeneous phase.

CuCl_2_ catalyzes the homolytic cleavage of two molecules of *t*-BuOOH into *t*-BuOO·, *t*-BuO· and H_2_O (Scheme 78a). *t*-BuO· radical abstracts a hydrogen atom from phthalan generating a benzyl radical (**129**). **129** reacts with *t*-BuOO· radical to form peroxide **130**, which loses *t*-BuOH to afford phthalide (Scheme 78b). Alternatively (Scheme 78c), **129** reacts with O_2_ (coming from either air or *t*-BuOOH reduction) and generates peroxide radical **131** which, after hydrogenation, releases water to generate phthalide. The addition of a radical scavenger (2,6-di-*tert*-butyl-4-methylphenol) inhibits the reaction, confirming the radicalic nature of mechanism. The effect of neocuproin can be explained by the equilibrium between catalytically active monomer and inactive dimer of copper complexes with bidentate bipyridyl ligands (Scheme 78d). At room temperature, the equilibrium favors the active monomeric form due to the steric effects of the methyl groups in positions 2 and 9; such effects are absent in phenanthroline and 4,7-dimethyl-1,10-phenanthroline, for which the equilibrium lies to the inactive dimer side.

#### 2.3.3. By Oxidation of 1,3-dihydroisobenzofuran in Heterogeneous Phase

**Scheme 79:**
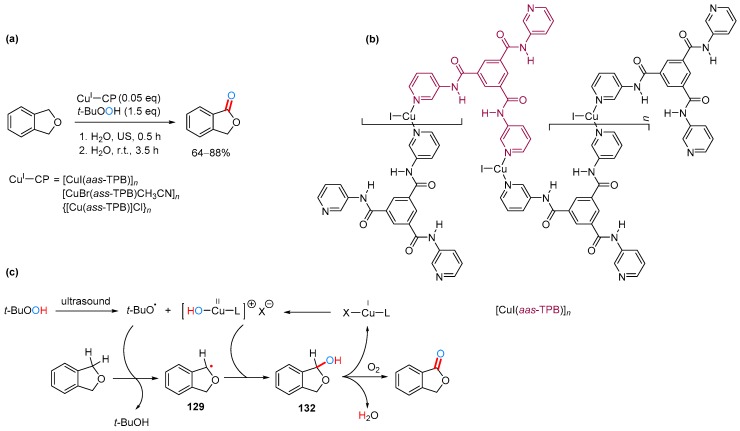
(**a**) Synthesis of phthalide from phthalan catalyzed by a Cu(I)–CP (CP=coordination polymer) complex. (**b**) Structure of the most active Cu(I)–CP catalyst. (**c**) Proposed mechanism.

The oxidation of phthalan can be performed in heterogenoeus phase by replacing CuCl_2_ with a coordination polymer of Cu(I): [CuI(*aas*-TPB)]*_n_*, [CuBr(*ass*-TPB)CH_3_CN]*_n_*, or {[Cu(*ass*-TPB)]Cl}*_n_* (TPB = *N*,*N*,*N*-*tris*(3-pyridinyl)-1,3,5-benzenetricarboxamide) (Scheme 79a,b) [63]. In this case, ultrasounds are required to generate *t*-BuO· radicals. The three catalysts can be used to oxidize several aryl alkanes to the respective ketones in allylic position. Heterogeneous catalysis is similar to homogeneous catalysis in terms of eco-friendliness, but has advantages and drawbacks in terms of practical applications. On one side, it provides full chemoselectivity because oxidation occurs only once, presumably due to the unique spatial confinement effect of coordination polymers; this selectivity results into much higher yields (64–88% *vs* 21% for phthalide). On the other side, the methodology in heterogeneous phase requires the tedious preparation of catalyst in three steps: (1) synthesis of ligand from 3-aminopyridine and 1,3,5-benzenetricarbonyl chloride [64], (2) heating (120 °C, 3 days) of metal salt and ligand in stainless steel containers in order to achieve high pressure, and (3) slow (5 °C/h) cooling down to room temperature to obtain the product in the crystalline form. 

Ultrasound treatment of *t*-BuOOH and X–Cu(I)–L polymer (X = halogen, L = TPB) generates *t*-BuO· radical and [HO–Cu(II)–L]^+^ X^−^ (Scheme 79c). Then, *t*-BuO· radical abstracts one of the allylic hydrogen atoms from the substrate to produce the allylic radical **129**, which further reacts with HO–Cu(II)LX to produce the corresponding secondary alcohol **132**. Finally, **132** is oxidized to ketone by O_2_. [CuI(*aas*-TPB)]*_n_* is the most active among the three catalysts for two reasons: it has the largest channels, which are needed to transport reactants and products; and it contains I^−^ anions, which may be easily cleaved generating the cationic Cu(II) complex.

## 3. α,β-Butenolides

α,β-Butenolides share an unsaturated lactone ring with phthalides. Therefore, in principle they can be prepared by the same methodologies used for the synthesis of phthalides, if vinyl carboxylic acids are employed in the place of benzoic acids. In practice, vinyl carboxylic acids react more slowly than benzoic acids in dehydrogenative coupling reactions that proceed by electrophilic substitution, as the positive charge built on the metallacycle during C—H activation cannot be delocalized. The lower reactivity of acrylic acids compared to benzoic acids in C—H bond activations partially explains why the methodologies available for the synthesis of α,β-butenolides are considerably lower in number than those for the preparation of phthalides.

### 3.1. γ-Substituted α,β-butenolides

#### γ-Alkyl-α,β-butenolides

**Scheme 80:**
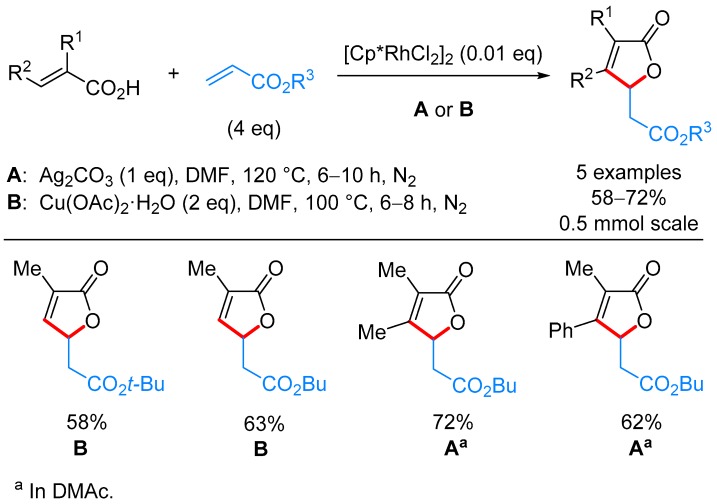
Rh-catalyzed annulative coupling of acrylic acids and acrylates for the synthesis of γ-alkyl-α,β-butenolides.

Acrylic acids, similarly to benzoic acids, undergo annulative coupling with acrylates under rhodium(III) catalysis to produce 3-substituted α,β-butenolides (Scheme 80) [65]. Oxidant and solvent effects are markedly substrate-dependent: methacrylic acid displays higher reactivity in the Cu(OAc)_2_·H_2_O/DMF system, whereas 2,3-dimethylacrilic acid and 2-methyl-3-phenylacryilic acid provide higher yields using Ag_2_CO_3_ in *N*,*N*-dimethylacetamide (DMAc).

**Scheme 81:**
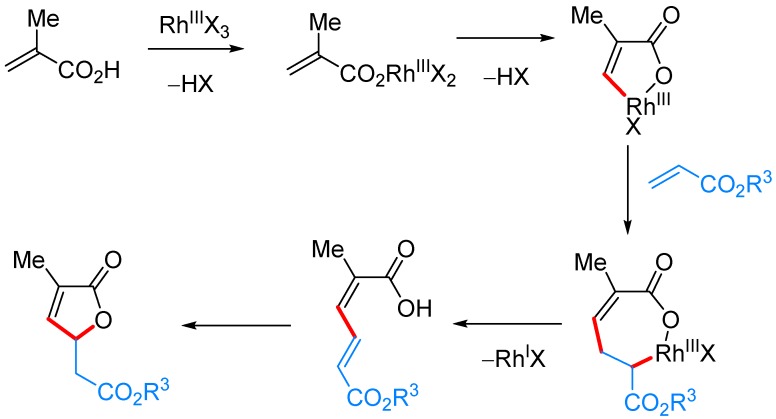
Proposed mechanism for the Rh-catalyzed annulative coupling of acrylic acids and acrylates.

The reaction likely proceeds through steps similar to those proposed for the annulative coupling of benzoic acids and alkenes, namely β-alkenylation followed by intramolecular Michael-type addition (Scheme 81).

### 3.2. γ,γ-Disubstituted α,β-butenolides

#### 3.2.1. γ-Alkyl-γ-vinyl-α,β-butenolides

**Scheme 82:**
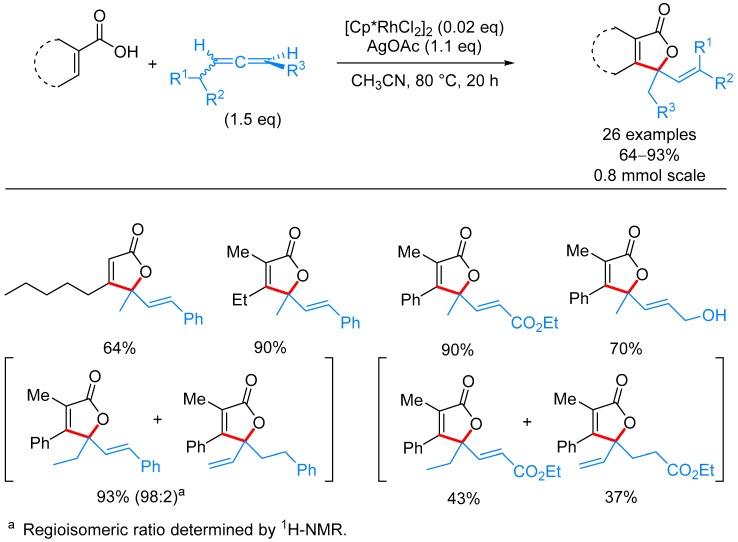
Rh-catalyzed annulative coupling of acrylic acids and allenes for the synthesis of γ-alkyl-γ-vinyl-α,β-butenolides.

Acrylic acids react with allenes analogously to benzoic acids, affording γ,γ-disubstituted α,β-butenolides (Scheme 82) [54]. The reaction requires slightly higher temperature (80 °C vs 60 °C) and a different silver oxidant (Ag_2_CO_3_ in the place of AgOAc) than in the case of benzoic acids. Acrylic acids bearing alkyl and aryl substituents at the positions α or β, or both, can be used in the reaction; however, no example with α-aryl substituted acids has been reported.

#### 3.2.2. γ-Alkylidenebutenolides

**Scheme 83:**
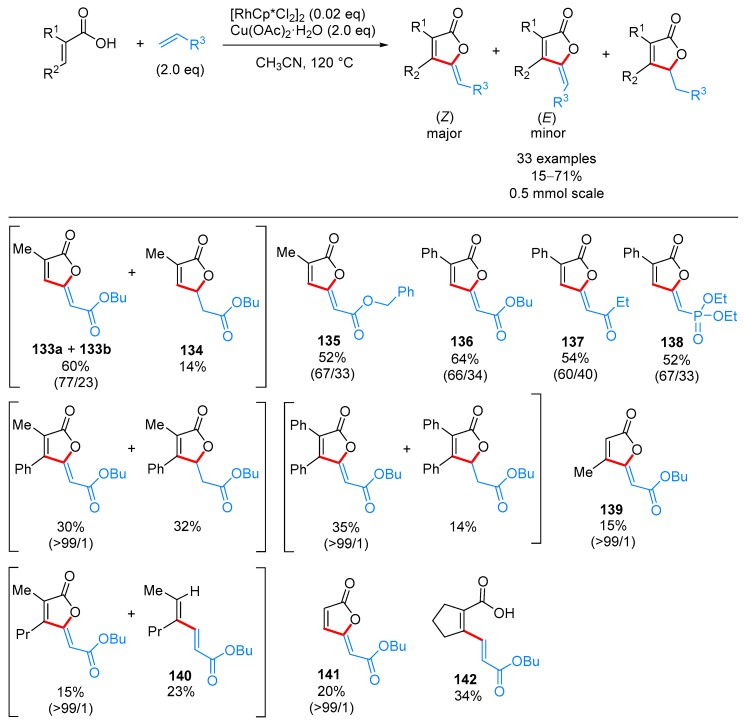
Rh-catalyzed annulative coupling of acrylic acids and acrylates for the synthesis of γ-alkylidenebutenolides.

When the reaction of acrylic acids and acrylates described above is performed at a higher temperature (120 °C) in CH_3_CN rather than DMF, γ-alkylidenebutenolides are obtained as the major product, alongside minor amounts of γ-alkylbutenolides (Scheme 83) [66]. Chemoselectivity (alkylbutenolide/alkylidenebutenolide ratio) and stereoselectivity (*Z*/*E* ratio) mainly depend on the substitution pattern of acid. α-Alkyl substituted acrylic acids are the least useful from a synthetic viewpoint, because they provide a mixture of three products (**133a**, **133b** and **134**), unless a sterically hindered ester is used (**135**). The introduction of an aromatic substituent in position α also prevents the formation of γ-alkylbutenolide, making the reaction fully chemoselective (**136–138**). α-Substituted acrylic always provide alkylidenebutenolides in a *Z*/*E* ratio ≥ 3:2. With α,β-disubstituted acrylic acids, the reaction is not chemoselective; however, the alkylidenebutenolides are obtained with full *Z* selectivity. Decarboxylation is observed with alkyl disubstituted acrylic acids (**140**). Only crotonic acid (which is β-substituted) and unsubstituted acrylic acid provide *Z*-alkylidenebutenolide as a single product (**139** and **141**, respectively). The reasons for such a selectivity trend are not clear. When the acid is part of a strained ring system, cyclization is prevented and the reaction stops at the alkenylation step, providing the *E* isomer exclusively (**142**). Various electrophilic alkenes (vinyl acrylates, vinyl ketones and vinyl phosphonates) can be used as coupling partners (**136–138**).

**Scheme 84:**
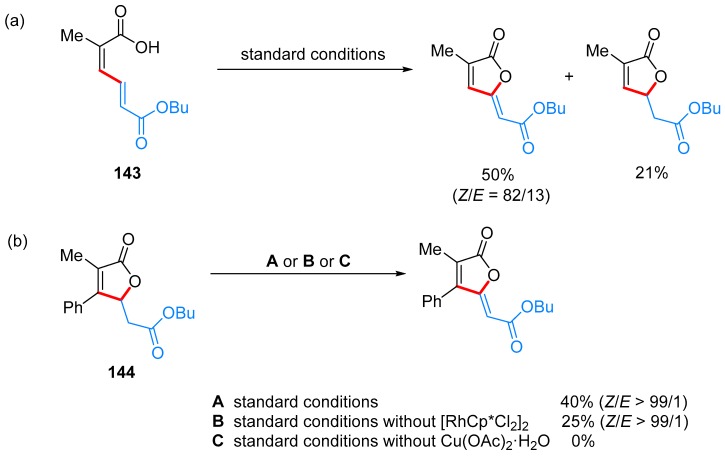
Control experiments for the Rh-catalyzed annulative coupling of acrylic acids and acrylates.

**Scheme 85:**
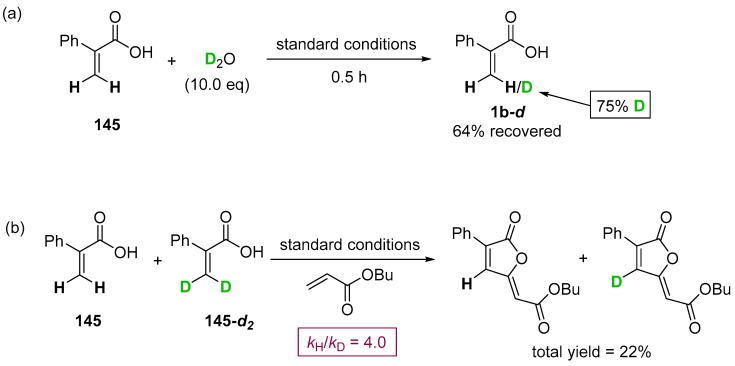
Deuterium-labelling experiments for the Rh-catalyzed annulative coupling of acrylic acids and acrylates.

The reaction likely follows a mechanism analogous to that described for benzoic acids (Scheme 48), in which γ-alkylbutenolides are formed first and then oxidized to γ-alkylidenebutenolides. Two pieces of evidence support this hypothesis (Scheme 84): (1) dienoic acid **143** (β-alkenylated intermediate) cyclizes under catalytic conditions to afford a mixture of γ-alkyl- and γ-alkylidenebutenolides (Scheme 84a); and (2) γ-alkylbutenolide **144** is partially converted into γ-alkylidenebutenolide in the presence of copper(II) oxidant but not in its absence (Scheme 84b). Additional mechanistic information is provided by deuterium-labelling experiments (Scheme 85). Treatment of 2-phenylacrylic acid (**145**) with deuterated water under standard conditions produces *Z*-selective olefinic H/D exchange, implicating a reversible cyclometalation (Scheme 85a). Furthermore, an intermolecular competition experiment between **145** and **145-*d*_2_** shows a kinetic isotope effect >1 (*k*_H_/*k*_D_ = 4.0), indicating a rate-determining C—H activation step (Scheme 85b).

**Scheme 86:**
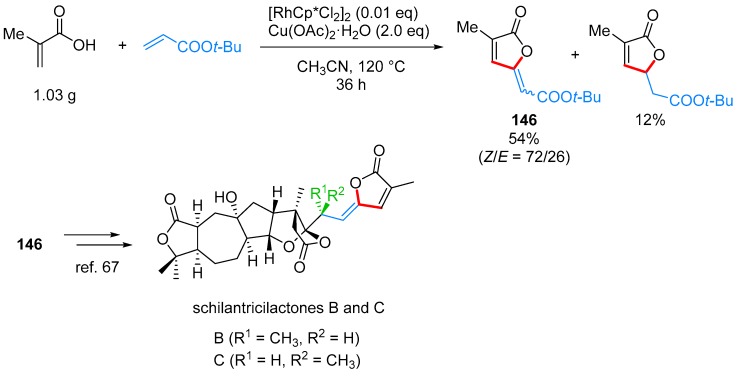
Gram-scale synthesis of a γ-alkylidenebutenolide intermediate in the preparation of schilantricilactones B and C.

The protocol has been applied to the gram-scale synthesis of an intermediate in the preparation of schilantricilactones B and C (Scheme 86) [67].

### 3.3. α,β-Disubstituted α,β-butenolides

**Scheme 87:**
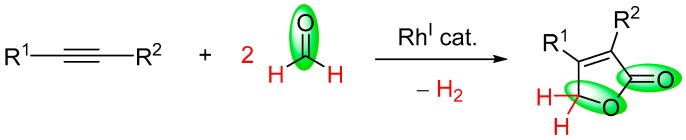
Dehydrogenative cyclohydrocarbonylation of alkynes affording α,β-butenolides.

The α,β-butenolide ring can be obtained by coupling an alkyne with two molecules of carbon monoxide and one molecule of hydrogen in a cyclohydrocarbonylation reaction. An eco-sustainable approach to this reaction involves the use of formaldehyde as a CO and H_2_ source (Scheme 87) [68].

**Scheme 88:**
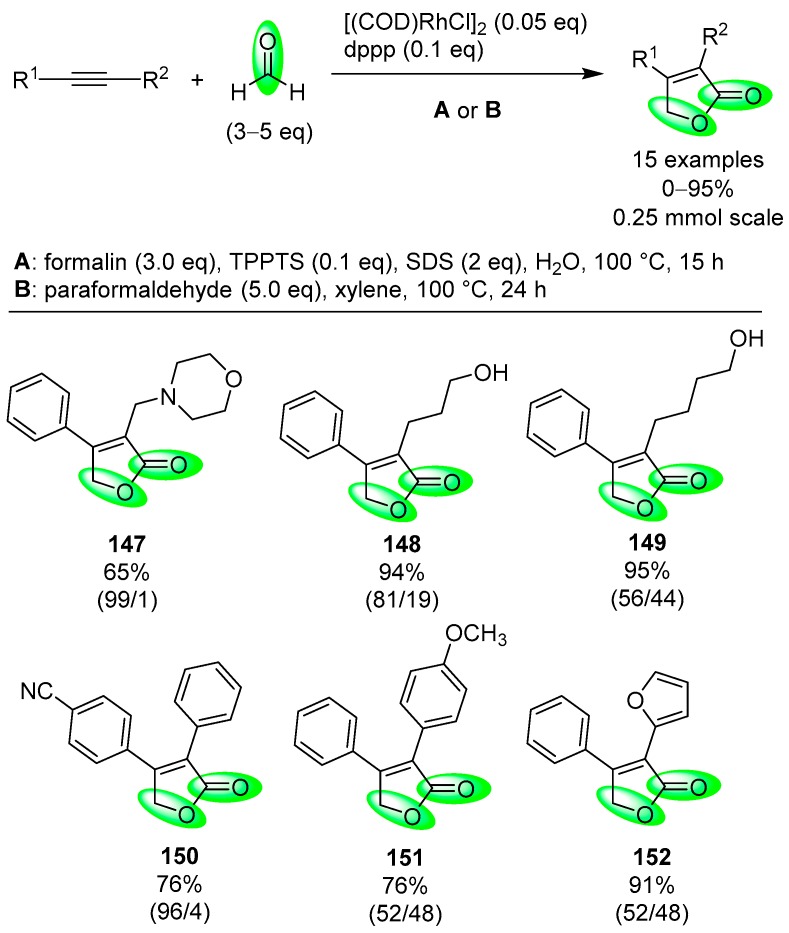
Rh-catalyzed cyclohydrocarbonyation of alkynes for the synthesis of α,β-disubstituted α,β-butenolides. TPPTS = triphenylphosphine-3,3′,3′′-trisulfonic acid trisodium salt; SDS = sodium dodecyl sulfate.

The reaction—catalyzed by Rh(I)—takes place in an aqueous surfactant (SDS, sodium dodecyl sulfate) at 100 °C and is accelerated by the use of an additional anionic surfactant (TPPTS, triphenylphosphine-3,3′,3′′-trisulfonic acid trisodium salt) in substoichiometric amounts (0.1 eq) (Scheme 88). This system provides a combined effect of metal and micellar catalysis. Two formaldehyde molecules are needed for the construction of the butenolide ring: one is incorporated as a carbonyl group, the other as a CH_2_O unit. Therefore, the reaction requires an excess of aldehyde (3–5 eq). Three pieces of evidence indicate that the hydrogens on the γ-carbon of α,β-butenolide are derived from formaldehyde rather than H_2_O: (1) the reaction of diphenylacetylene also takes place in non-aqueous solvents such as xylene (using paraformaldehyde in the place of formaldehyde); (2) no product is formed if gaseous CO is used in the place of formaldehyde; and (3) the product is also formed in the absence of HCHO under an atmosphere of CO—H_2_ (1:1), although in low (6%) yield. This methodology, exploiting formaldehyde as a CO source, offers the advantage of avoiding the use of toxic gaseous CO. However, it only works with internal alkynes, as terminal alkynes such as phenylacetylene undergo self-polymerization in the presence of Rh(I). Groups suitable for further functionalization (OH, phthaloyl) are tolerated on alkyne. Moderate to excellent regioselectivities (63:37–99:1) are observed for unsymmetrically substituted alkynes. With *mono*-aryl alkynes, the aromatic ring always ends up at the β-position in the product (**147–149**). With *di*-aryl alkynes, the β-position of butenolide is occupied by the most electron-poor aromatic ring (**150–152**). The reasons for such a regioselectivity are unclear.

**Scheme 89:**
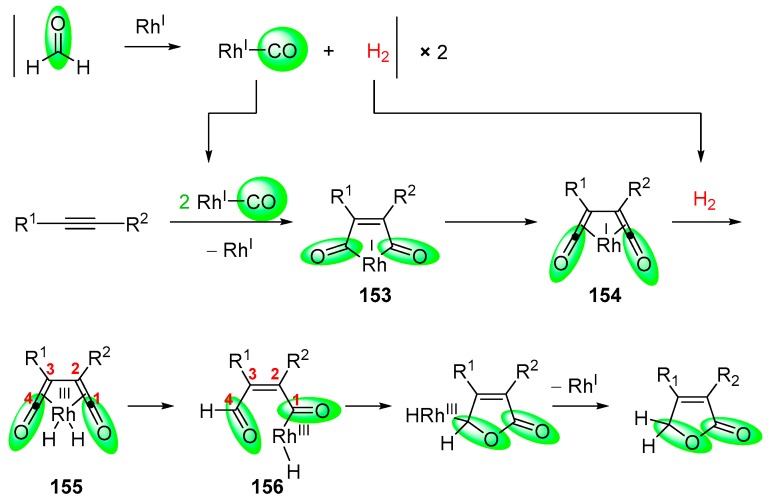
Proposed mechanism for the Rh-catalyzed cyclohydrocarbonyation of alkynes.

In the proposed mechanism (Scheme 89), formaldehyde undergoes decarbonylation to afford a Rh^I^—CO complex and H_2_. Cyclization of alkyne with two carbonyl moieties on rhodium produces maleoylrhodium **153**, which isomerizes to (η^4^-bisketene)rhodium **154**. The oxidative addition of H_2_, followed by 1,4-addition of Rh—H bond in **155**, produces the β-formyl-acylrhodium intermediate **156**. The addition of acyl—Rh bond to the formyl group in **156**, followed by reductive elimination, gives the α,β-butenolide.

## 4. Conclusions

Over 30 methods for the direct or semi-direct synthesis of phthalides and α,β-butenolides have been reported so far, mostly developed over the past ten years (Table 2). 

The following conclusions can be made:

(1) All the reactions are catalyzed by a transition metal (usually Rh^I^, Rh^III^, or Ru^II^); most of them take place in homogeneous phase and follow an ionic mechanism.
(2) Oxidative cyclizations require the use of an oxidant, either metallic (Cu^II^ or Ag^I^) or organic (*t*-BuOOH); O_2_ can be used as the final oxidant in a few cases.
(3) Phthalides are easier to obtain than α,β-butenolides due to the presence of phenyl ring, which assists the C—H activation step.
(4) Several methodologies have been developed for the direct or semi-direct synthesis of 3-substituted phthalides; only a few are available for the preparation of 3,3-disubstituted phthalides and α,β-butenolides; none have been reported for the synthesis of spirophthalides or polymeric phthalides. Furthermore, there is no direct method available to introduce two fully saturated substituents in position 3. Phthalide disubstitution in position 3 through direct methods is made it difficult by the limited availability of unsaturated alkynes and allenes. Isolation from natural sources and classical multi-step synthesis are currently the only alternative for phtalides and butenolides that cannot be obtained by direct methods.
(5) Despite the advantage provided in terms of sustainability, most methodologies still suffer from several limitations: (a) the use of expensive, non-recyclable catalysts (such as Rh or Pd), which compromises their application on a large scale; (b) the use of metal oxidants, which generate waste undermining the sustainability of the reaction; (c) the lack of enantioselectivity (except for ketone hydroacylation); and (d) the relatively small diversity of products. The reaction scope is quite large in terms of number (with over 20 examples of products reported in most cases), but the methodologies have usually been applied to the synthesis of simple structures rather than large drug-like molecules. Naturally occurring, reactive functional groups suitable for further functionalization, such as OH and NH_2_, are usually not tolerated on the coupling partners.
(6) The development of new methods for the synthesis of heterocycles (including phthalides and butenolides) via C—H bond functionalization is made challenging by two aspects: (a) the large number of factors (substrate, catalyst, oxidant, additives, solvent, as well as reagents ratio and concentration) that affect chemo- and regio-selectivity and are difficult to control; and (b) the paucity of comprehensive mechanistic studies, which are necessary to fully rationalize the results. The available mechanistic studies mostly consist of deuterium-labelling experiments and control experiments that focus on the C—H bond activation step and the identification of some intermediates. The exact nature of the active species as well as the role of oxidant, additive, solvent, and metal ligand are still poorly understood despite the extensive screening that is performed to optimize the reaction conditions. In particular, it is unclear whether the metal oxidant may contribute to catalysis, especially considering the fact that it is used in large (100–1000-fold) excess with respect to catalyst. The isolation and characterization of reactive intermediates would shed light on the mechanism, helping the design of chiral ligands and the development of analogous reactions.

## Figures and Tables

**Table 1:**
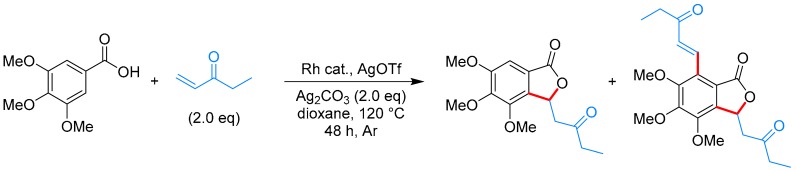
Catalyst and additive effects on chemoselectivity of the Rh-catalyzed annulative coupling of benzoic acids and alkenes.

Cat.	eq. AgOTf	Additive	NMR Yield (%)	
[Cp*RhCl_2_]_2_ (0.08 eq)	0.40	-	23	48
[Cp*Rh(MeCN)_3_](SbF_6_)_2_ (0.16 eq)	-	-	30	27
[(COD)RhCl]_2_ (0.08 eq)	0.24	Ph_4_CpH_2_ (0.16 eq)	51	22
[(COD)RhCl]_2_ (0.08 eq)	0.24	Me_5_CpH (0.16 eq)	21	20

**Table 2:** Summary of preparation methods of phthalides and α,β-butenolides.

Product	Coupling Partners		Conditions	Ref.
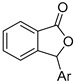	ArCOOH	Ar’CHO	[Cp*RhCl_2_]_2_/AgOTf, Ag_2_CO_3_, dioxane, 150 °C	[20]
	ArCHO	Ar’CHO	[Cp*RhCl_2_]_2_, AgBF_4_, (4-CF_3_)C_6_H_4_NH_2_, Ag_2_CO_3_, diglyme, 90 °C	[22]
	ArC(=NOMe)OMe	Ar’CHO	[Cp*RhCl_2_]_2_, AgSbF_6_, DCE, 110 °C	[23]
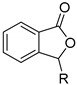	ArCOOH	alkene	[RuCl_2_(*p*-cymene)]_2_, Cu(OAc)_2_·H_2_O, H_2_O, 80 °C	[24]
			[RuCl_2_(*p*-cymene)]_2_, Cu(OAc)_2_·H_2_O, PEG-400/H_2_O, 80 °C	[25]
			[RuCl_2_(*p*-cymene)]_2_, Cu(OAc)_2_·H_2_O, CH_3_CN, 100 °C	[26]
			[RuCl_2_(*p*-cymene)]_2_, O_2_, MOAc/ROH, 60–80 °C	[27]
			[RuCl_2_(*p*-cymene)]_2_, AcOH, O_2_, GVL, 80 °C	[28]
			[Cp*RhCl_2_]_2_, NaClO_2_, AcOH, H_2_O, 120 °C	[29]
			[(COD)RhCl]_2_, AgOTf, DCPD, Cu(OAc)_2_·H_2_O, PhCl, 120 °C	[30]
			[Cp*RhCl_2_]_2_, O_2_, KHCO_3_, AcOH, DCE, 60 °C	[31]
			Pd(OAc)_2_, Cu(OAc)_2_·H_2_O, DMF, 120 °C	[33]
			[Cp*RhCl_2_]_2_, Cu(OAc)_2_·H_2_O, *o*-xylene, 120 °C	[34]
	ArCONR_2_	alkene	[Cp*RhCl_2_]_2_, Cu(OAc)_2_·H_2_O, DCE/AcOH, 130 °C	[35]
	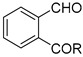	–	RuHCl(CO)(PPh_3_)_3_, PhCH_3_, 90 °C	[39]
			[(COD)RhCl]_2_, AgNO_3_, duanphos, PhCH_3_, 75–100 °C	[40]
			CoCl_2_, (*R*,*R*)-Ph-BPE, In, CH_3_CN	[41]
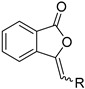	ArCOOH	alkene	[Cp*RhCl_2_]_2_, Cu_2_O, Ag_2_CO_3_, PhCH_3_, 105 °C	[45]
	ArCOOH	vinyl arene	Pd(OAc)_2_, Cu(OAc)_2_·H_2_O, DMF, 100 °C	[33]
			Pd(OAc)_2_, Ag_2_O, DMF, 110 °C	[49]
	ArCOOH	vinyl acetate	[Cp*RhCl_2_]_2_, CuO, LiCl, KI, PhCH_3_/*t*-AmOH, 120 °C	[50]
	ArCOOH	alkyne	[Cp*RhCl_2_]_2_, AgSbF_6_, Ag_2_O, PivOH, *o*-xylene, 100 °C	[51]
	ArCOOH	anhydride	[(COD)RhCl]_2_, CsF, NMP, 155 °C	[52]
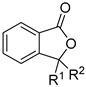	ArCOOH	allene	[Cp*RhCl_2_]_2_, AgOAc, CH_3_CN, 60 °C	[54]
	ArCOOH	alkyne	Ru/ZrO_2_, KOAc, mesitylene, 170 °C	[55]
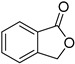	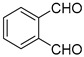	–	HRh(CO)(PPh_3_)_3_, EtOH, 80 °C	[56]
			RhCl_3_·3H_2_O, PPh_3_, Na_2_CO_3_, EtOH, 80 °C	[56]
			RhH(PPh_3_)_3_, PhH, 19 °C	[57]
			Et_4_N^+^[(μ-H)Cr_2_(CO)_10_]^−^	[58]
			[Rh(dcpe)]_2_ClO_4_, acetone, 34 °C	[60]
	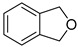	–	CuCl_2_, neocuproin, *t*-BuOOH, H_2_O, r.t.	[61]
			CuI(*aas*-TPB)]*_n_*, *t*-BuOOH, H_2_O, r.t.	[63]
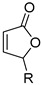	acrylic acid	acrylate	Ag_2_CO_3_, DMF, 120 °C	[65]
			Cu(OAc)_2_·H_2_O, DMF, 100 °C	[65]
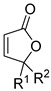	acrylic acid	allene	[Cp*RhCl_2_]_2_, AgOAc, CH_3_CN, 80 °C	[54]
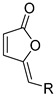	acrylic acid	acrylate	[Cp*RhCl_2_]_2_, Cu(OAc)_2_·H_2_O, CH_3_CN, 120 °C	[66]
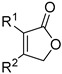	alkyne	HCHO	[(COD)RhCl]_2_, dppp, TPPTS, SDS, H_2_O, 100 °C	[68]
		(CH_2_O)*_n_*	[(COD)RhCl]_2_, dppp, xylene, 100 °C	[68]
